# *Pax9* and *Gbx2* Interact in the Pharyngeal Endoderm to Control Cardiovascular Development

**DOI:** 10.3390/jcdd7020020

**Published:** 2020-05-25

**Authors:** Catherine A. Stothard, Silvia Mazzotta, Arjun Vyas, Jurgen E. Schneider, Timothy J. Mohun, Deborah J. Henderson, Helen M. Phillips, Simon D. Bamforth

**Affiliations:** 1 Newcastle University Biosciences Institute, Centre for Life, Newcastle-upon-Tyne NE1 3BZ, UK; catherinestothard@gmail.com (C.A.S.); silvia.mazzotta@abdn.ac.uk (S.M.); A.Vyas1@newcastle.ac.uk (A.V.); deborah.henderson@newcastle.ac.uk (D.J.H.); helen.phillips@newcastle.ac.uk (H.M.P.); 2 Biomedical Imaging, University of Leeds, Leeds LS2 9JT, UK; J.E.Schneider@leeds.ac.uk; 3 The Francis Crick Institute, London NW1 1AT, UK; tim.mohun@gmail.com

**Keywords:** *Pax9*, *Gbx2*, *Tbx1*, pharyngeal endoderm, arch arteries

## Abstract

The correct formation of the aortic arch arteries depends on a coordinated and regulated gene expression profile within the tissues of the pharyngeal arches. Perturbation of the gene regulatory networks in these tissues results in congenital heart defects affecting the arch arteries and the outflow tract of the heart. Aberrant development of these structures leads to interruption of the aortic arch and double outlet right ventricle, abnormalities that are a leading cause of morbidity in 22q11 Deletion Syndrome (DS) patients. We have recently shown that *Pax9* functionally interacts with the 22q11DS gene *Tbx1* in the pharyngeal endoderm for 4th pharyngeal arch artery morphogenesis, with double heterozygous mice dying at birth with interrupted aortic arch. Mice lacking *Pax9* die perinatally with complex cardiovascular defects and in this study we sought to validate further potential genetic interacting partners of *Pax9*, focussing on *Gbx2* which is down-regulated in the pharyngeal endoderm of *Pax9*-null embryos. Here, we describe the *Gbx2-*null cardiovascular phenotype and demonstrate a genetic interaction between *Gbx2* and *Pax9* in the pharyngeal endoderm during cardiovascular development.

## 1. Introduction

Normal morphogenesis of the mammalian heart and aortic arch arteries is controlled by a complex interaction of tissues and gene expression. Genetic mutations that alter heart development result in congenital heart defects which affect nearly 1% of the population, and result in structural defects to the heart and its associated great vessels [1]. Conotruncal defects affecting the outflow tract (OFT) of the heart and the aortic arch arteries are a major feature in 22q11 deletion syndrome (DS) [2], and of the 45 genes haploinsufficient in these patients, *TBX1* is thought to be the most important for cardiovascular development [3,4,5]. The OFT is formed from cells of the second heart field (SHF), a group of progenitor cells located in the splanchnic mesoderm that migrate to the heart and subsequently continue to provide progenitors to the heart throughout embryogenesis [6]. Initially formed as a common vessel, the OFT divides into the aorta and pulmonary trunk, and these two vessels separate and rotate around each other as the aorticopulmonary septal complex spirals caudally. Septation is completed by the fusion of the interventricular septum with the OFT endocardial cushions of the atrioventricular canal [7] and requires a contribution from neural crest cells [8]. Failure in the fusion of the OFT cushions, or if the OFT rotates aberrantly, can result in common arterial trunk (CAT) and transposition of the great arteries (TGA), respectively [9]. An atrioventricular septal defect (AVSD) can occur if there is an abnormal fusion of the superior and inferior endocardial cushions [10]. The aortic arch arteries are derived from the pharyngeal arch arteries (PAAs) that form within the pharyngeal arches from SHF-derived endothelial cells [11]. The pharyngeal arches are a transient series of protrusions that develop in a cranial to caudal sequence along the lateral surface of the head [12] and are comprised of endoderm, mesoderm, ectoderm, and neural crest cell-derived mesenchyme [13]. The pharyngeal arches develop by forming pouches from the endoderm that meet clefts from the ectoderm to define the boundaries of each arch [14] and the endoderm is considered vital for providing cues for this patterning event in a range of species [15,16,17]. Disruption to pharyngeal segmentation is seen when pharyngeal endoderm gene expression is perturbed [15,18,19,20]. There are five bilaterally symmetrical pairs of PAAs that connect the aortic sac to the dorsal aorta. The PAAs then asymmetrically remodel to form the left-sided aortic arch and double circulatory system [21] and this process is conserved between mice and humans [22]. Aberrant PAA formation and remodelling can result in defects such as interruption of the aortic arch type B (IAA-B) [23].

The 22q11DS gene *Tbx1* is known to interact with many genes in cardiovascular development, including *Pax9* [24,25] and *Gbx2* [24]. *Pax9* is specifically expressed in the pharyngeal endoderm at embryonic day (E) 9.5 [26], although it is later expressed in the craniofacial region and skeleton. Mice deficient for *Pax9* die perinatally with a cleft palate, absent pharyngeal-derived glands, skeletal abnormalities and have complex heart and aortic arch artery defects [25,27]. *Pax9* has been shown to functionally interact with *Tbx1* in the pharyngeal endoderm for 4th PAA morphogenesis, as double-heterozygous mice develop IAA-B [25]. *Gbx2* has also been implicated in cardiovascular development [28,29] and is normally expressed in all three germ layers of the mouse at the early head fold stage (E7.5) in a domain that extends rostrally from the posterior end of the embryo, encompassing the node, into the prospective hindbrain [30]. In the pharynx, *Gbx2* is expressed in the pharyngeal arch endoderm and ectoderm at E8.5 [31] but restricted to the pharyngeal endoderm by E9.5 [28]. *Gbx2* is downregulated in the pharyngeal endoderm of *Pax9*-null embryos [25] and in the pharyngeal endoderm and ectoderm of *Tbx1*-null embryos [28].

In this study, we have examined the cardiovascular phenotype in *Gbx2*-null mice and identified a genetic interaction for cardiovascular development between *Gbx2* and *Pax9* in complex mutants in the pharyngeal endoderm. We also explored a potential genetic interaction between *Tbx1*, *Pax9* and *Gbx2* for aortic arch artery morphogenesis.

## 2. Materials and Methods

### 2.1. Mice

The mice used in this study have previously been described: *Gbx2^flox^* [32], *Pax9^+/−^* [27], *Pax9^Cre^* [25], *Sox2^Cre^* [33], *Tbx1^+/−^* [34], and *R26R^eYFP^* [35]. All mice were maintained on a C57Bl/6J genetic background. All studies involving animals were performed in accordance with the UK Home Office Animals (Scientific Procedures) Act 1986.

### 2.2. Breeding

Male and female mice were mated and the detection of a vaginal plug the next morning considered to be embryonic day (E) 0.5. Pregnant females were culled on the required day and embryos collected. Embryos at E9.5–E11.5 were staged by somite counting. PCR genotyping primers are available on request.

### 2.3. Imaging

Magnetic resonance imaging (MRI), High Resolution Episcopic Microscopy (HREM) and micro-computed tomography (µCT) techniques were performed as previously described [25,36,37,38,39]. Volume data sets were segmented using Amira software (ThermoFisher Scientific, Waltham, MA, USA) to create 3-dimensional (3-D) images. Structures were manually outlined using the label field function of Amira and surface rendered to produce the 3-D images. Intra-cardiac ink injections were performed as described [28]. Haematoxylin and eosin staining, immunohistochemistry, and whole-mount in situ hybridisation, were performed using standard techniques. mRNA expression on sections was examined by in situ hybridisation using RNAscope^®^ Multiplex Fluorescent v2 Assay (Advanced Cell Diagnostics, Newark, CA, USA) following the manufacturer’s instructions. Probe and antibody details are given in Table S5.

### 2.4. Identifying Conserved Binding Sites in the GBX2 Locus

Human and mouse genomic *GBX2* sequences were aligned in Geneious 10.2.6 (https://www.geneious.com) using the Align feature, identifying a highly conserved region ~2kb downstream from the 2nd and final *GBX2* coding exon. This region was scanned for TBX1/5 and PAX5/9 consensus binding sequences using the rVista (https://rvista.dcode.org/) and JASPAR (http://jaspar.genereg.net/) databases. One TBX5 and three PAX5/9 sites were identified with the following sequences: TBX-BE-1, CAGCACCTCA; PAX-BE-1, AGGCAGCCCTTGATG; PAX-BE-2, GTCTCAGCTGGCTGATTAA; and PAX-BE-3, TTAATCCAAGGTTGGGTTTTG.

### 2.5. Luciferase Assay

The 500 bp Gbx2 conserved region was amplified from mouse genomic DNA by PCR and sub-cloned into the pGL3-Promoter plasmid (Promega, Madison, WI, USA) downstream of luciferase. The cDNAs for *PAX9* and *TBX1* were cloned into pcDNA3.1. JEG3 cells were seeded at a density of 1 × 10^5^ cells per well in 24-well plates and transfections were performed in triplicate with jetPRIME (Polyplus-transfection, Illkirch, France) using a total of 0.5 µg DNA in a reaction volume of 300 μL. Positive control reporter constructs were 2xTkGl2, containing synthetic TBX binding sites [5], and p2.4BMP4-luc which binds PAX9 [40]. A renilla luciferase vector (5 ng) was included as a transfection control. Wells without DNA or with the empty pcDNA3.1 vector served as negative controls. All wells received an equal quantity of DNA per transfection. A dual-luciferase assay (Promega) was performed 24 h after transfection.

### 2.6. Flow Cytometry

The pharyngeal arch region of E9.5 *Pax9Cre;eYFP* control embryos (n = 5; 23–27 somites) was dissected from the rest of the embryo and dissociated to single cells with Accumax (ThermoFisher Scientific) by incubating at 37 °C for 30 min. The reaction was stopped by the addition of 10% fetal calf serum (FCS), the cells washed in PBS, and resuspended in 10% FCS. Cells were stained with propidium iodide. Fluorescence-activated cell sorting was performed on a Becton Dickinson FACS Aria II using a 100 µm nozzle and a sheath pressure of 20 psi. Single cells were gated using FSC-A vs SSC-A followed by FSC-A vs. FSC-H and FSC-A vs SSC-W to remove any doublets. Live single cells were gated using propidium iodide vs. FSC-A, and this population finally gated on eYFP-positive and -negative cells and sorted into collection tubes.

### 2.7. Quantitative Real-Time RT-PCR (qPCR)

RNA from flow-sorted cells was extracted using Trizol reagent (ThermoFisher Scientific) combined with a Purelink RNA Mini Kit (ThermoFisher Scientific) and on-column DNase1 treatment. RNA was eluted with 30 µL RNase-free water. Total RNA was converted to cDNA using a High-Capacity cDNA Reverse transcription kit (Applied Biosystems) and random hexamers. qPCR was performed using SYBR Green JumpStart Taq ReadyMix (Sigma, St. Louis, MO, USA) using previously described *Gbx2* and *Gapdh* primers [25]. All qPCR reactions were performed in triplicate on a QuantStudio 7 Real-Time PCR System (ThermoFisher Scientific). Data were analysed using the comparative Ct method.

### 2.8. Statistical Analysis

A chi-squared test was used to compare genotype frequencies of litters. Fisher’s exact test and Pearson chi-squared test for associations were used to compare defect frequencies between the different genotypes (SPSS). qPCR data were tested for variance using the Shapiro–Wilk test (Prism 8.01 software, GraphPad) and a two-tailed unpaired t-test performed. Luciferase data were analysed using one-way ANOVA with Tukey’s multiple comparison test (Prism 8.01 software, GraphPad). Groups were considered significantly different when *p* < 0.05.

## 3. Results

### 3.1. Gbx2 Expression in the Pharyngeal Endoderm

**Figure 1:**
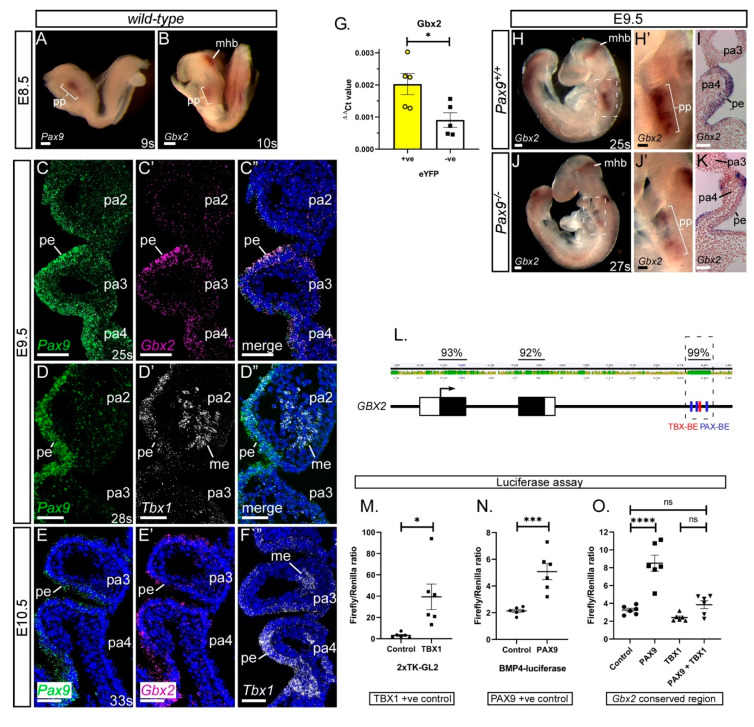
*Gbx2* and *Pax9* expression in the pharyngeal endoderm. (**A**–**F**) RNA in situ hybridisation was used to visualise transcripts in the normal developing embryo. At E8.5 whole embryo in situ hybridisation reveals *Pax9* (**A**) and *Gbx2* (**B**) expression in the pharyngeal pouch (pp) region. *Gbx2* is also expressed in the mid-hindbrain (mhb) (n = 3 embryos per probe, 9–11 somites). (**C**–**F**) RNAScope probes were used on sections at E9.5 (n = 3, 24–28 somites) and E10.5 (n = 3, 33 somites). At E9.5 *Pax9* and *Gbx2* (**C**), and *Pax9* and *Tbx1* (**D**) co-localise to the pharyngeal endoderm. At E10.5 *Pax9* (**E**), *Gbx2* (**E’**) and *Tbx1* (**F**), are all expressed in the pharyngeal endoderm. (**G**) qPCR on flow-sorted cells from *Pax9Cre;eYFP* E9.5 embryos (n = 5, 23–27 somites) shows a significant enrichment for *Gbx2* in the eYFP-positive cells compared to eYFP-negative. * *p* < 0.05; two-tailed unpaired t-test. (**H**,**J**) Whole embryo in situ hybridisation at E9.5 in wild-type (**H**; n = 3, 24–26 somites) and *Pax9^−/−^* (**J**; n = 6, 23–27 somites) embryos. *Gbx2* expression is reduced in the pharyngeal pouch in *Pax9^−/−^* embryos. (**I**,**K**) Sections of stained embryos revealed reduced *Gbx2* staining in the pharyngeal endoderm in *Pax9^−/−^* embryos (**K**) compared to controls (**I**). (**L**) Diagram of the *GBX2* locus showing the degree of conservation (%) between human and mouse *GBX2* sequences. A 500 bp region, ~2kb down from the second, and final, *GBX2* coding exon is 99% conserved and contains PAX (blue) and TBX (red) binding elements (BE). (**M**–**O**) Luciferase assays show that Pax9 activates the *Gbx2* conserved region but Tbx1 does not (**O**). Controls for Tbx1 (**M**) and Pax9 (**N**) show significant activation of luciferase. Data presented as the mean ± s.e.m. of 6 individual experiments, each performed in triplicate. * *p* < 0.05, *** *p* < 0.01, **** *p* < 0.001. Scale bars: 100 μm in (**A**,**B**,**H**,**J**); 50 μm in (**C**–**F**),(**H’**, **I**, **J’**,**K**). Abbreviations: me, mesoderm; pa, pharyngeal arch; pe, pharyngeal endoderm; s, somites. The somite numbers given in the legend reflect the range analysed for the whole study. The figure contains representative images only.

Coordinated gene expression in the pharyngeal arches is a prerequisite for the correct development of the PAA. We confirmed the expression of *Gbx2*, *Pax9* and *Tbx1* in mid-embryogenesis mouse embryos. At E8.5, *Gbx2* and *Pax9* are expressed in the pharyngeal region of the mouse embryo, with *Gbx2* also expressed at the mid-hindbrain boundary (Figure 1A,B) [27,28,41]. At E9.5, *Gbx2* and *Pax9* are co-expressed in the pharyngeal endoderm (Figure 1C), as are *Pax9* and *Tbx1* (Figure 1D) [25]. *Gbx2*, *Pax9* and *Tbx1* expression are maintained in the pharyngeal endoderm at E10.5 (Figure 1E,F). Analysis of *Pax9Cre* activated eYFP positive cells [25] by qPCR from E9.5 embryos confirmed that *Gbx2* expression is enriched in the pharyngeal endoderm compared to the remaining pharyngeal arch tissues (Figure 1G). *Gbx2* expression is bilaterally reduced in the pharyngeal endoderm of *Pax9*-null embryos at E9.5 as shown by whole-mount in situ hybridisation (Figure 1H–K) [25].

We have previously identified *Gbx2* as a potential target gene of *Pax9* [25]. To examine the *Gbx2* locus for potential regulatory elements we aligned the mouse and human sequences and found a 500 bp region with 99% homology (Figure 1L). This region, located ~2kb downstream of the second and final *Gbx2* coding exon, contains a previously reported TBX binding site [42]. Scanning this region identified the known TBX site as well as three potential PAX binding sites. This 500 bp conserved region was sub-cloned into a luciferase plasmid and transfected into JEG3 cells with PAX9 and TBX1 expression plasmids. Dual-luciferase assays showed a significant upregulation of luciferase in the presence of the PAX9 expression plasmid, but no effect was seen with TBX1 (Figure 1O). Co-transfection of PAX9 with TBX1 resulted in no increase in luciferase compared to the control suggesting that TBX1 might be repressing the effect of PAX9 on the *Gbx2* conserved region. This data implies that PAX9 and TBX1 may interact with a potential *GBX2* enhancer to regulate the expression of *GBX2*, and this interaction is likely to occur in the pharyngeal endoderm where all three genes are expressed from E9.5.

### 3.2. Gbx2-Null Cardiovascular Defects

As *Gbx2* was significantly down-regulated in *Pax9^−/−^* embryos [25], and PAX9 was able to activate a conserved region of the *Gbx2* locus, we asked whether these two genes genetically interacted in vivo. *Gbx2^−/−^* mice have previously been shown to have cardiovascular defects at foetal and embryonic stages [28,29]. To confirm these observations we created a null allele of *Gbx2* by crossing *Gbx2^flox^* mice [32] with *Sox2Cre* mice [33], which drives Cre-mediated recombination in all embryonic tissues by gastrulation. *Gbx2^flox^;Sox2Cre* mice, carrying the recombined *Gbx2*-null allele, hereafter referred to as the *Gbx2^−^* allele, were subsequently crossed with wild-type mice to breed out the *Sox2Cre* allele and to propagate the *Gbx2^−^* allele through the germline. Heterozygous *Gbx2^+/−^* mice were intercrossed to generate *Gbx2^−/−^* embryos for analysis of cardiovascular developmental defects. Slide in situ hybridisation on E9.5 embryo sections demonstrated complete loss of *Gbx2* RNA in *Gbx2^−/−^* embryos (Figure S1). Genotype analysis of embryos and neonates (E8.5 to P0) indicated there was a statistically significant reduction in the expected number of *Gbx2^−/−^* offspring observed (Table S1) despite no evidence of an excessive number of resorptions.

**Figure 2:**
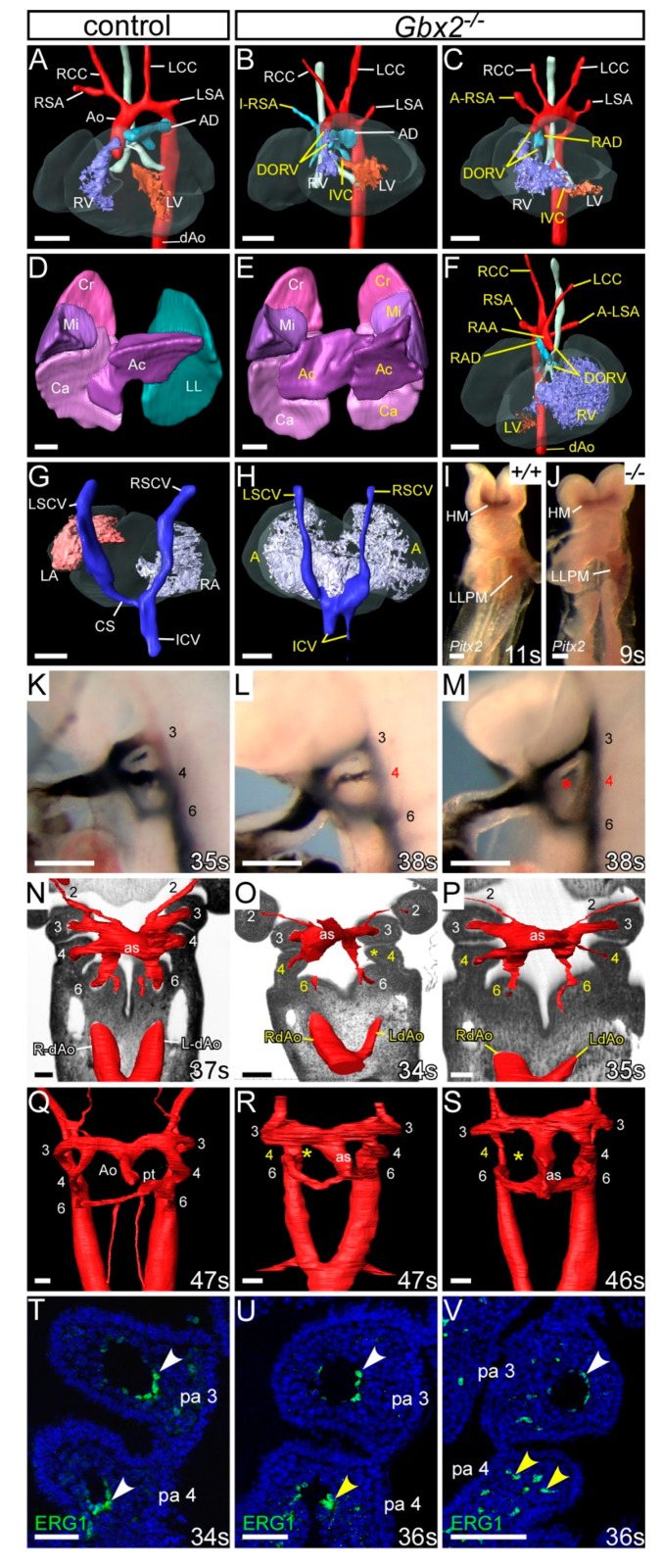
Cardiovascular defects in *Gbx2^−/−^* embryos. (**A**–**H**) 3D reconstructions from MRI datasets of E15.5 embryos. (**A**) *Gbx2^+/+^* control embryo with normal heart and aortic arch arteries. (**B**,**C**) In *Gbx2^−/−^* embryos (n = 25), defects seen include double outlet right ventricle (DORV) with interventricular communication (IVC), retro-oesophageal aberrant right subclavian artery (A-RSA), isolated RSA (I-RSA) and right-sided arterial duct (RAD). (**D**–**H**) Left-right patterning defects were observed in a subset of *Gbx2^−/−^* embryos (n = 9/25). (**D**) Normal lungs from a control embryo showing cranial (Cr), middle (Mi), caudal (Ca) and accessory (Ac) lobes of the right lung, and a single lobed left lung (LL). (**E**) *Gbx2^−/−^* embryo with right pulmonary isomerism. (**F**) *Gbx2^−/−^* embryo heart with mirror image arrangement of the arch arteries and the ventricles, DORV and an aberrant left subclavian artery (A-LSA). (**G**,**H**) Dorsal view of the heart and the caval veins. (**G**) Control embryo showing the right superior caval vein (RSCV) and inferior caval vein (ICV) draining directly into the right atrium (RA) and the left SCV (LSVC) draining into the RA via the coronary sinus (CS). (**H**) *Gbx2^−/−^* embryo with the right and left SCVs, and the paired ICV, draining directly into a common atrium (right atrial isomerism). (**I**,**J**) Whole-mount in situ hybridisation with a *Pitx2* riboprobe. *Pitx2* isoforms were seen in the head mesoderm (HM) and left lateral plate mesoderm (LLPM) of control (n = 3) and *Gbx2^−/−^* (n = 6) embryos. All embryos were between 9–11 somites. (**K**–**M**) Intracardiac ink injection into E10.5 embryos (34–38 somites). (**K**) In control embryos (n = 18), PAAs 3–6 are patent to ink, are of equivalent diameter and are bilaterally symmetrical. (**L**,**M**) In *Gbx2^−/−^* embryos (n = 17), the 4th PAAs are frequently hypoplastic (**L**) or non-patent to ink (**M**; asterisk). (**N**–**S**) Embryos were examined by high-resolution episcopic microscopy at E10.5 (34–35 somites; **N**–**P**) and E11.5 (45–47 somites; **Q**–**S**). Coronal views are shown. (**N**) In control E10.5 embryos (n = 5), the 3rd, 4th, and 6th PAAs, and the left and right dorsal aortae (L/RdAo), are of equal size and bilaterally symmetrical. (**O**,**P**) In *Gbx2^−/−^* embryos (n = 3), the 4th PAAs were either absent (**O**; asterisk), or hypoplastic (**O**,**P**). The left dorsal aorta was seen to be abnormally thinner than the right (**O**,**P**). (**Q**) In control embryos at E11.5 (n = 3), the outflow tract is septated into the aorta (A) and pulmonary trunk (pt), and the right 6th PAA has thinned. (**R**,**S**) In *Gbx2^−/−^* embryos (n = 5), the 4th PAAs are frequently unilaterally absent (asterisk). Septation of the aortic sac (as) is delayed. (**T**–**V**) Immunostaining using anti-ERG1 antibody at E10.5 (34–36 somites). (**T**) Control embryos (n = 3) have a ring of ERG1-positive endothelium lining the 3rd and 4th PAAs (white arrowheads). (**U**,**V**) In *Gbx2^−/−^* embryos (n = 4), the 4th PAAs appeared to have abnormal endothelial cells (**U**), or disorganized endothelial cells within the 4th pharyngeal arch in the absence of a PAA (**V**; yellow arrowheads). Scale bars: 500 μm in (**A**–**H**); 100 μm in (**I**–**S**); 50 μm in (**T**–**V**). Abbreviations: Ao, aorta; AD, arterial duct; LCC, left common carotid artery; LSA, left subclavian artery; LV, left ventricle; pa, pharyngeal arch; RCC, right common carotid artery; RSA, right subclavian artery; RV, right ventricle; s, somites. The somite numbers given in the legend reflect the range analysed for the whole study. The figure contains representative images only.

Analysis of *Gbx2^−/−^* embryos at E15.5 by magnetic resonance imaging (MRI) revealed that 64% (n = 16/25) had some form of cardiovascular developmental defect including aberrant right subclavian artery (A-RSA), right-sided aortic arch (RAA), right-sided arterial duct (RAD) and double-outlet right ventricle (DORV) (Figure 2A–C; Table 1), although interruption of the aortic arch (IAA) was not observed. Of these *Gbx2^−/−^* embryos with cardiovascular defects, 56% (n = 9/16) displayed additional overt left-right patterning defects such as right pulmonary isomerism, bilateral superior caval veins draining into a common right atrium (i.e., right atrial isomerism), and mirror-image aortic arch arteries (Figure 2D–H; Table 1). An enlargement of the right dorsal aorta, compared to the left, was also noted in two *Gbx2^−/−^* embryos analysed by high-resolution episcopic microscopy (HREM), indicating that these embryos would have developed an aberrant right-sided dorsal aorta by the foetal stage (Figure 2O,P). A left-right patterning phenotype in *Gbx2^−/−^* mice has not previously been reported. Left-right patterning is established via a *Nodal-Pitx2c* gene regulated pathway [43] and to investigate whether the left-right patterning defect was due to altered *Pitx2c* expression, whole-mount in situ hybridisation was performed on *Gbx2^−/−^* embryos at E8.5 (n = 6). No obvious change in *Pitx2c* expression, however, was observed in the left lateral plate mesoderm (Figure 2I,J).

Analysis of *Gbx2^−/−^* embryos at E10.5 by intracardiac ink injection and HREM demonstrated that the 4th PAA was affected in 71% (n = 12/17) of embryos, being either absent or hypoplastic (Figure 2K–P; Table 2). Ink injection of *Gbx2^+/−^* embryos at E10.5 revealed a low level of unilateral 4th PAA defects (Table 2). Embryos at E11.5 also showed PAA defects by HREM including absent and hypoplastic 4th PAAs as well as delayed septation of the OFT compared to stage-matched controls (Figure 2Q–S). Immunohistochemical staining for ERG1 showed that the endothelium within the 3rd and 4th pharyngeal arches had formed lumenised PAAs at E10.5 in control embryos (Figure 2T), but in *Gbx2^−/−^* embryos a visibly reduced number of endothelial cells around the 4th PAA, or only isolated endothelial cells present within the 4th pharyngeal arch, were observed (Figure 2U,V).

All mice deficient for *Gbx2* die perinatally, and a high proportion of these are with cardiovascular abnormalities affecting the outflow tract and/or the 4th PAA-derived arteries. In contrast to previously published data, where 50% of *Gbx2*-null embryos had a PAA phenotype and 39% of foetuses had a cardiovascular defect [28,29], we observed a higher penetrance of cardiovascular abnormalities at embryonic (71%) and foetal (64%) stages. Our *Gbx2*-null embryos did not present with IAA as previously described [29]. *Gbx2* has been postulated to be a downstream target of *Pax9* [25], and here we also demonstrate that a conserved region in the *Gbx2* locus is activated by PAX9.

### 3.3. Genetic Interaction between Gbx2 and Pax9

**Figure 3:**
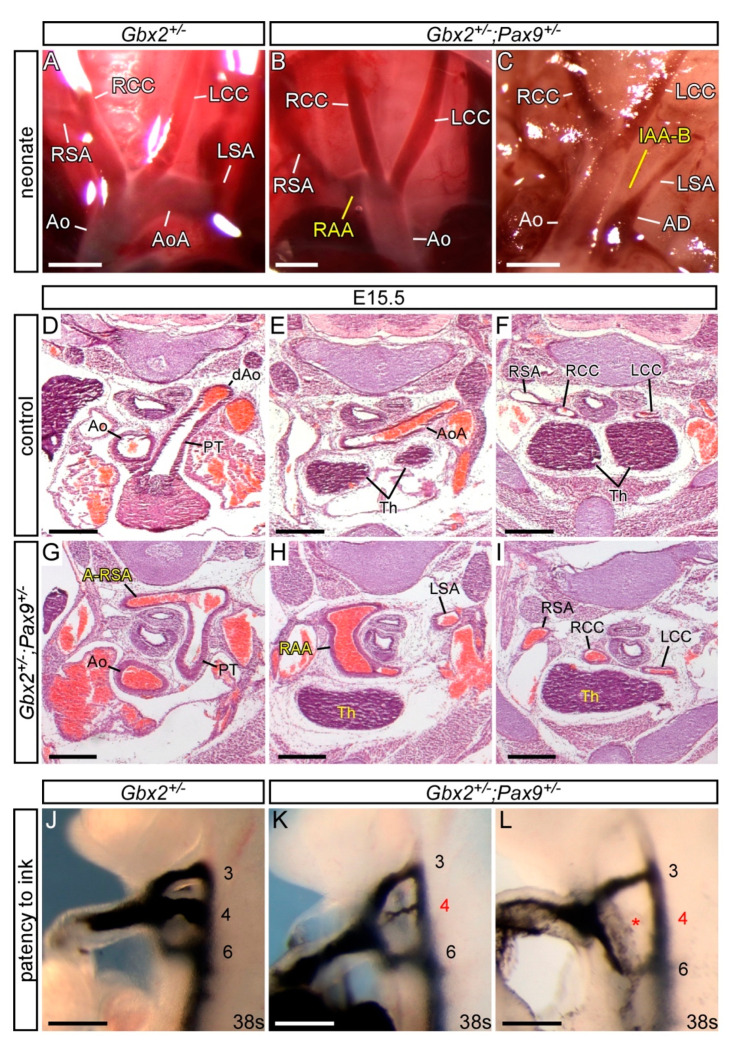
Cardiovascular defects in *Gbx2^+/−^;Pax9^+/−^* mice. (**A**–**C**) Neonates were dissected on the day of birth. (**A**) Arch arteries of control neonates were normal. (**B**) A subset of *Gbx2^+/−^;Pax9^+/−^* neonates (4/12) displayed right-sided aortic arch (RAA; **B**) or IAA-B (**C**). (**D**–**I**) H&E stained transverse sections of E15.5 embryos. (**D**–**F**) Normal outflow tract (**D**), aortic arch arteries and thymus (Th; **E**,**F**) in a control embryo. (**G**–**I**) A *Gbx2^+/−^;Pax9^+/−^* embryo (from n = 16) with aberrant right subclavian artery (A-RSA; **G**), a right-sided aortic arch (RAA; **H**) and abnormal thymus (**H**,**I**). (**J**–**L**) Intracardiac ink injection into E10.5 embryos (34–41 somites). (**J**) In control embryos, PAAs 3–6 are patent to ink, are of equivalent diameter and are bilaterally symmetrical. (**K**,**L**) In *Gbx2^+/−^;Pax9^+/−^* embryos (n = 63), 32% had hypoplastic (**K**) or non-patent to ink (**L**; asterisk) 4th PAAs. Scale bars: 1 mm in (**A**–**C**); 500 μm in (**D**–**L**). Abbreviations: AD, arterial duct; Ao, aorta; AoA, aortic arch; LCC, left common carotid artery; LSA, left subclavian artery; PT, pulmonary trunk; RCC, right common carotid artery; RSA, right subclavian artery; s, somites. The somite numbers given in the legend reflect the range analysed for the whole study. The figure contains representative images only.

To investigate a genetic interaction in vivo, mice heterozygous for *Pax9* and *Gbx2*, which are viable and fertile with no observed developmental defects, were intercrossed to produce *Gbx2^+/−^;Pax9^+/−^* mice and embryos for analysis. *Gbx2^+/−^;Pax9^+/−^* mice were observed to survive to weaning albeit at a significantly lower than expected number (*p* < 0.05; Table S2). To investigate any perinatal lethality to explain the loss of *Gbx2^+/−^;Pax9^+/−^* mice, neonates were observed from the day of birth. Four pups were recovered which had died on the first post-natal day, all of which were *Gbx2^+/−^;Pax9^+/−^*, and showed a cardiovascular defect when dissected including RAA, IAA-B and A-RSA (Figure 3A–C; Table 1). Surviving littermates were culled and a further eight *Gbx2^+/−^;Pax9^+/−^* neonates were identified which showed no cardiovascular defects. All wild type (n = 8), *Gbx2^+/−^* (n = 10) and *Pax9^+/−^* (n = 9) littermates were normal. To further investigate the *Gbx2^+/−^;Pax9^+/−^* cardiovascular phenotype, embryos were collected at E15.5 and analysed by histology. From 16 *Gbx2^+/−^;Pax9^+/−^* embryos collected, two had a defect affecting the arch arteries including RAA and A-RSA (Figure 3D–I) and RAD (Table 1). Embryos were collected at E10.5 to analyse the forming PAA by ink injection. This revealed that 30% (n = 19/63) of *Gbx2^+/−^;Pax9^+/−^* mutants had a defect where the 4th PAA was either hypoplastic or absent in affected embryos (Figure 3J–L; Table 2).

Our data therefore indicates that there is a genetic interaction occurring between *Gbx2* and *Pax9*, albeit at a low incidence, where biallelic expression of both genes is required for cardiovascular development to proceed normally. There was a loss in the expected number of double heterozygous mice at weaning and an observable aortic arch artery phenotype in a subset of embryos and neonates, including IAA-B which was not seen in *Gbx2^−/−^* mice in this study (Table 1).

**Figure 4:**
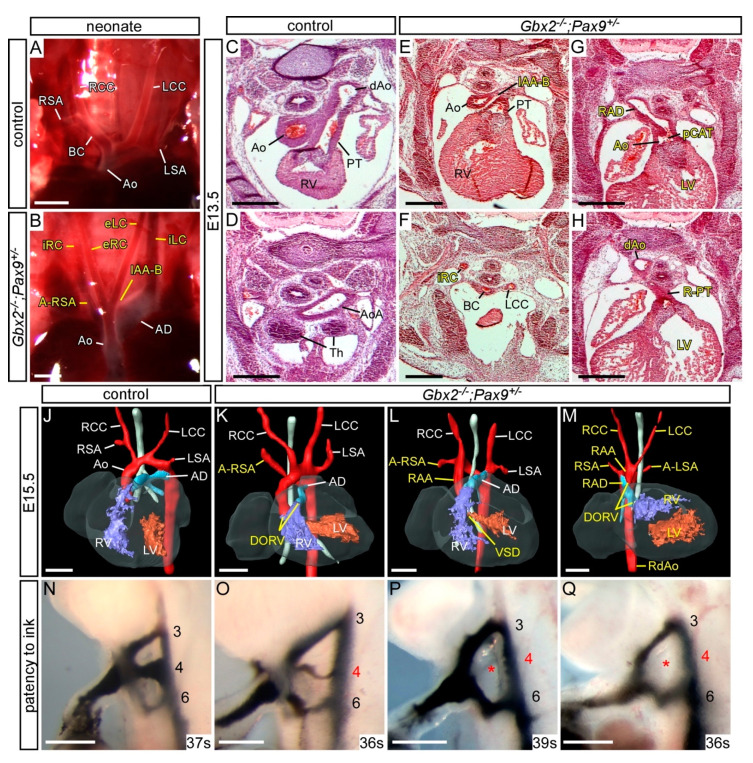
Cardiovascular defects in *Gbx2^−/−^;Pax9^+/−^* mice. (**A**–**B**) Neonates were dissected on the day of birth. (**A**) Arch arteries of control neonates were normal. (**B**) One *Gbx2^−/−^;Pax9^+/−^* neonate found dead on the day of birth displayed IAA-B and an aberrant left and right internal and external carotid artery (iLC, eLC, iRC, eRC). (**C**–**H**) H&E stained transverse sections of E13.5 embryos (n = 5). A normal outflow tract (**C**), aortic arch (Ao) and thymus (Th; **D**) are seen in control embryos. In two *Gbx2^−/−^;Pax9^+/−^* mutant embryos (**E**–**H**) the defects include IAA-B (**E**), an aberrant right internal carotid artery (iRC; **F**), and a partial common arterial trunk (pCAT) emerging from the left ventricle (**G**). The pulmonary trunk is right-sided (R-PT; **H**) and the aorta was interrupted (not shown). The thymus is absent in both mutants (**F**,**H**). (**J**–**M**) 3D reconstructions of MRI datasets of hearts from a normal control embryo (**J**) and *Gbx2^−/−^;Pax9^+/−^* mutant embryos (n = 8). Defects include aberrant right subclavian artery (A-RSA; **K**,**L**), double outlet right ventricle (DORV; **K**,**M**), right-sided aortic arch (RAA; **L**,**M**). Situs patterning defects were also observed, including mirror image aortic arch arteries (RAA, RAD, RdAo) with aberrant left subclavian artery (A-LSA) and aberrantly positioned ventricles (**M**). (**N**–**Q**) Intracardiac ink injection into E10.5 embryos (35–39 somites). (**N**) In control embryos, PAAs 3–6 are patent to ink. (**K**,**L**) In *Gbx2^+/−^;Pax9^+/−^* embryos (n = 7), all had an aberrant 4th PAA including hypoplastic (**O**) or non-patent to ink (**P**,**Q**; asterisk) vessels. Scale bars: 1 mm in (**A**–**B**); 500 μm in (**C**–**Q**). Abbreviations: Ao, aorta; AD, arterial duct; dAo, dorsal aorta; LCC, left common carotid artery; LSA, left subclavian artery; LV, left ventricle; PT, pulmonary trunk; RCC, right common carotid artery; RSA, right subclavian artery; RV, right ventricle; s, somites. The somite numbers given in the legend reflect the range analysed for the whole study. The figure contains representative images only.

To see if a further reduction in *Gbx2* and *Pax9* alleles would result in a more penetrant cardiovascular phenotype, *Gbx2^+/−^;Pax9^+/−^* mice were intercrossed and embryos and neonates collected for analysis. Genotyping revealed that all mice null for *Gbx2* were under-represented (*p* = 1.12 × 10^−4^; Table S3). We first analysed mutant mice null for *Gbx2* and simultaneously heterozygous for *Pax9* (i.e., *Gbx2^−/−^;Pax9^+/−^*). One *Gbx2^−/−^;Pax9^+/−^* neonate was recovered that died on the day of birth with IAA-B, A-RSA, and an absent left common carotid artery resulting in the left internal and external carotid arteries arising from the aortic arch and dorsal aorta as previously described in *Pax9^−/−^* embryos [25] (Figure 4A,B). *Gbx2^−/−^;Pax9^+/−^* embryos at E13.5 and E15.5 were examined for cardiovascular defects by histology and MRI respectively, which showed a significantly increased penetrance of OFT and arch artery defects compared to those observed in *Gbx2^−/−^* mice (100% versus 64%, *p* < 0.05; Figure 4C–M; Table 1). There was also a significant increase in the presentation of IAA-B in *Gbx2^−/−^;Pax9^+/−^* mice compared to *Gbx2^−/−^* mice (36% versus 0, *p* < 0.01; Figure 4B,E; Table 1), a defect not observed in our *Gbx2^−/−^* or *Pax9^+/−^* mice. We also observed a partial CAT (type A4 where the common trunk is associated with IAA [44]) in one *Gbx2^−/−^;Pax9^+/−^* embryo (Figure 4G), a phenotype not previously described in *Gbx2*-null and *Pax9*-null embryos [25,28,29]. Analysis of the PAA at E10.5 by ink injection revealed that all *Gbx2^−/−^;Pax9^+/−^* embryos had an affected 4th PAA, with a significantly higher incidence of bilateral 4th PAA defects compared to *Gbx2^−/−^* embryos (*p* < 0.05; Figure 4N–Q; Table 2).

**Figure 5:**
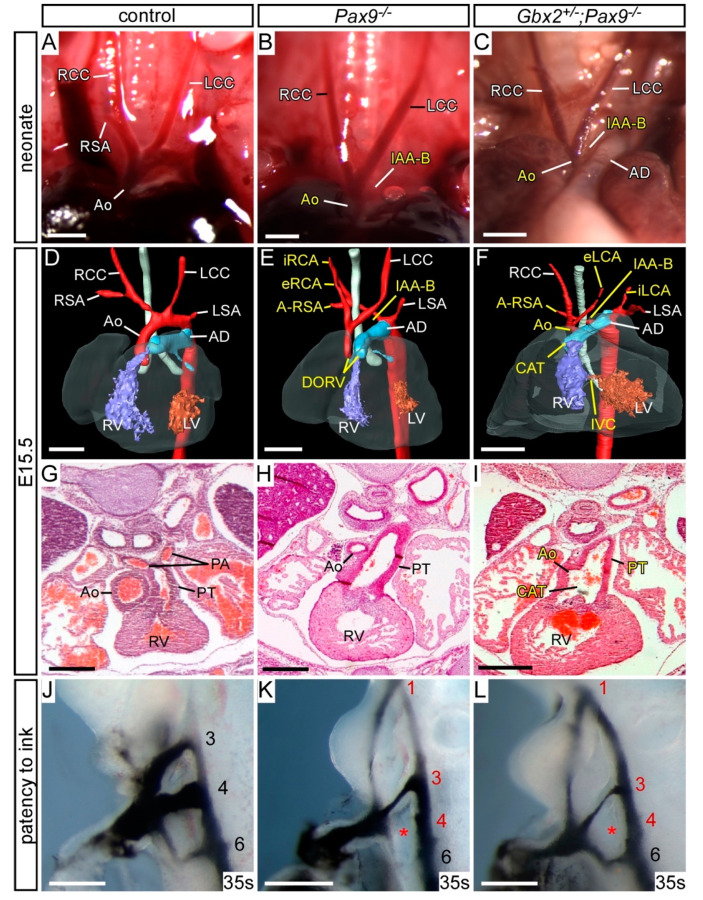
Cardiovascular defects in *Gbx2^+/−^;Pax9^−/−^* mice. (**A**–**C**) Neonates were dissected on the day of birth. (**A**) Arch arteries of control neonates were normal. (**B**) *Pax9^−/−^* (n = 2) and (**C**) *Gbx2^−/−^;Pax9^+/−^* (n = 2) neonates found dead on the day of birth displayed interruption of the aortic arch (IAA-B) and presumed aberrant right subclavian artery. (**D**–**F**) 3D reconstructions of E15.5 hearts from MRI datasets. (**D**) *Gbx2^+/+^* control embryo with normal heart and aortic arch arteries. (**E**) *Pax9^−/−^* embryos (n = 7) presented with defects such as IAA-B, A-RSA, double outlet right ventricle (DORV), hypoplastic aorta and aberrant carotid arteries (eRCA, iRCA). (**F**) *Gbx2^+/−^;Pax9^−/−^* embryos (n = 7) showed typical *Pax9^−/−^* cardiovascular defects including IAA-B and absent common carotid artery, but also common arterial trunk (CAT). (**G**–**I**) E15.5 embryos were sectioned and stained with H&E. (**G**) Control embryos had a normal outflow tract. (**H**) *Pax9^−/−^* embryos had DORV (not shown) and hypolastic aorta. (**I**) *Gbx2^+/−^;Pax9^−/−^* embryo with CAT. (**J**–**L**) Embryos at E10.5 were analysed by intracardiac ink injection (35–38 somites). (**J**) Control embryos had normal PAAs patent to ink. (**K**) *Pax9^−/−^* embryos showed typical defects such as persistent 1st, hypoplastic 3rd and absent 4th PAAs (asterisk), as did *Gbx2^+/−^;Pax9^−/−^* embryos (n = 6; **L**). Scale bars: 1 mm in (**A**–**C**); 500 μm in (**D–L**). Abbreviations: Ao, aorta; AD, arterial duct; LCC, left common carotid artery; LSA, left subclavian artery; LV, left ventricle; PA, pulmonary artery; PT, pulmonary trunk; RCC, right common carotid artery; RSA, right subclavian artery; RV, right ventricle; s, somites. The somite numbers given in the legend reflect the range analysed for the whole study. The figure contains representative images only.

As *Pax9* heterozygosity modified the *Gbx2^−/−^* phenotype we next looked to see if *Gbx2* heterozygosity modified the *Pax9^−/−^* phenotype. *Gbx2^+/−^;Pax9^−/−^* embryos examined at E15.5 and E10.5 presented with the typical *Pax9*-null cardiovascular phenotype (Figure 5) and no left-right patterning defects were seen. Two cases of type A4 partial CAT, however, were observed (Figure 5I; Table 1). At E10.5 there was an insignificant increase in the penetrance of 1^st^ and 3rd PAA defects in the *Gbx2^+/−^;Pax9^−/−^* embryos (Figure 5L; Table 2).

**Figure 6:**
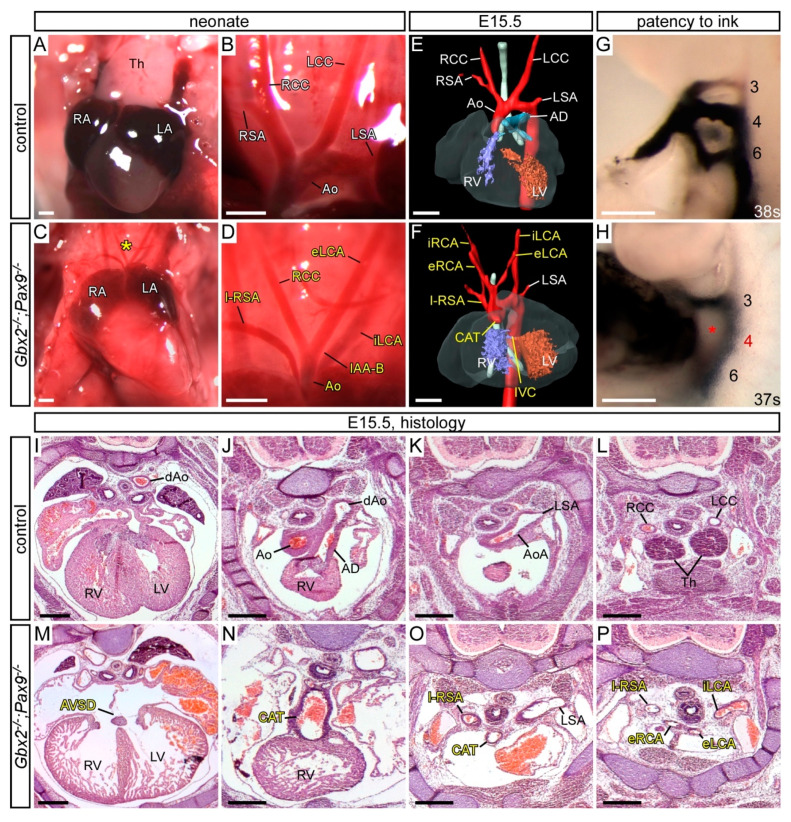
Cardiovascular defects in the three *Gbx2^−/−^;Pax9^−/−^* mutants recovered. (**A**–**D**) Neonates were dissected on the day of birth. The thymus (**A**) and arch arteries (**B**) were normal in controls, but in a *Gbx2^−/−^;Pax9^−/−^* neonate the thymus was absent (asterisk; **C**) and the arch arteries were abnormal (**D**) displaying interrupted aortic arch type B (IAA-B), isolated right subclavian artery (I-RSA) and abnormal left carotid arteries (eLCA, iLCA). (**E**,**F**) 3D reconstructions of E15.5 hearts from MRI datasets. (**E**) Control embryo with normal heart and aortic arch arteries. (**F**) *Gbx2^−/−^;Pax9^−/−^* embryo with IAA-B, I-RSA, common arterial trunk (CAT) with interventricular communication (IVC), and aberrant carotid arteries (eLCA, iLCA, eRCA, iRCA). (**G,H**) Embryos at E10.5 were analysed by intracardiac ink injection. (**G**) Control embryos had normal PAAs patent to ink. (**H**) *Gbx2^−/−^;Pax9^−/−^* embryo with a non-patent 4th PAA (asterisk). (**I**–**P**) H&E stained transverse sections of E15.5 embryos. (**I**–**L**) Control embryo with normal heart (**I**), outflow tract (**J**), aortic arch arteries (**K**,**L**) and thymus (Th; **L**). (**M**–**P**) Sections from the *Gbx2^−/−^;Pax9^−/−^* embryo depicted in **F**. (**M**) An atrioventricular septal defect (AVSD) is seen, as well as the CAT (**N**) with associated I-RSA (**O**) and aberrant carotid arteries (**P**). Scale bars: 500 μm. Abbreviations: AD, arterial duct; Ao, aorta; AoA, aortic arch; dAo, dorsal aorta; LA, left atrium; LCC, left common carotid artery; LSA, left subclavian artery; LV, left ventricle; RA, right atrium; RCC, right common carotid artery; RSA, right subclavian artery; RV, right ventricle.

From the *Gbx2^+/−^;Pax9^+/−^* intercross only three *Gbx2^−/−^;Pax9^−/−^* mice were recovered from a total of 172 offspring, one each at P0, E15.5 and E10.5, and these mice displayed the typical *Pax9^−/−^* phenotypes, as well as AVSD and full CAT (with an isolated right subclavian artery), both of which are defects not previously seen in either *Gbx2^−/−^* or *Pax9^−/−^* embryos (Figure 6; Table 1 and Table 2).

The thymus is always absent in *Pax9^−/−^* embryos [25], yet usually unaffected in *Gbx2^−/−^* embryos (Figure S2; Table S4). In 70% of *Gbx2^−/−^;Pax9^+/−^* embryos the thymus was abnormal or absent. The palate, however, was only affected in mice with *Pax9^−/−^* genotypes (Figure S2).

It appears, therefore, that creating complex alleles of *Gbx2* and *Pax9* reveals evidence of a genetic interaction occurring between these two genes as cardiovascular defects are seen in double heterozygous embryos. Furthermore, by creating more complex *Gbx2;Pax9* alleles such as *Pax9* heterozygosity with the *Gbx2^−/−^* genotype and *Gbx2* heterozygosity with the *Pax9^−/−^* genotype, not only are *Pax9*-null type defects such as IAA-B and absent common carotid arteries introduced, but also a CAT phenotype not seen in either single *Gbx2^−/−^* or *Pax9^−/−^* embryos is observed.

### 3.4. Gbx2 and Pax9 Interact in the Pharyngeal Endoderm for Cardiovascular Development

**Figure 7:**
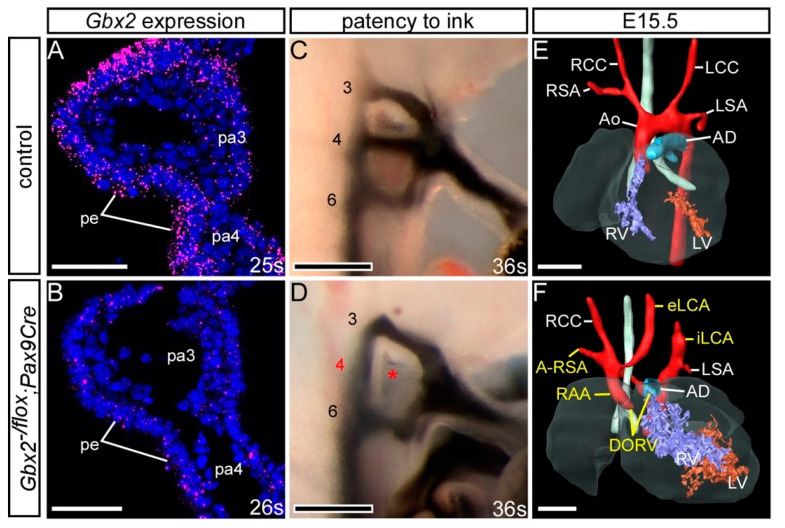
Conditional deletion of *Gbx2* from the pharyngeal endoderm. Embryos with one *Gbx2*-null allele and a heterozygous conditional deletion of *Gbx2* using *Pax9Cre* (*Gbx2^−/flox^;Pax9Cre*) were analysed. (**A**,**B**) RNAscope in situ hybridisation for *Gbx2* at E9.5 (n = 5 per genotype; 24–29 somites). (**A**) In control embryos *Gbx2* expression was observed in the pharyngeal endoderm (pe) of the pharyngeal arches (pa). (**B**) *Gbx2* expression was reduced in the pharyngeal endoderm of *Gbx2^−/flox^;Pax9Cre* mutants. (**C**,**D**) Embryos at E10.5 were analysed by intracardiac ink injection (36–38 somites). (**C**) Control embryos had normal PAAs patent to ink. (**D**) *Gbx2^−/flox^;Pax9Cre* embryos (n = 14) showed PAA defects such as absent 4th PAAs (asterisk). (**E**,**F**) 3D reconstructions of E15.5 hearts from MRI datasets. (**E**) Control embryo with normal heart and aortic arch arteries. (**F**) A *Gbx2^−/flox^;Pax9Cre* embryo (from n = 14 analysed) with double-outlet right ventricle (DORV), right-sided aortic arch (RAA), aberrant right subclavian artery (A-RSA) and abnormal left external and internal carotid arteries (eLCA, iLCA). Scale bars: 50 μm in (**A**,**B**); 100 μm in (**C**,**D**); 500 μm in (**E**,**F**). Abbreviations: Ao, aorta; AD, arterial duct; LCC, left common carotid artery; LSA, left subclavian artery; LV, left ventricle; RCC, right common carotid artery; RSA, right subclavian artery; RV, right ventricle; s, somite. The somite numbers given in the legend reflect the range analysed for the whole study. The figure contains representative images only.

To further investigate the site of this genetic interaction we utilised our *Pax9Cre* mouse line, where the *Pax9Cre* allele corresponds to a *Pax9*-null allele [25], to conditionally recombine the *Gbx2^flox^* allele specifically in the pharyngeal endoderm (Figure S3). Embryos conditionally deleted for *Gbx2* using *Pax9Cre* (i.e., *Gbx2^−/flox^;Pax9Cre*) lost the majority of *Gbx2* mRNA expression from the pharyngeal endoderm at E9.5 (Figure 7B). Embryos at E10.5 were examined by ink injection to assess the PAAs. Whereas only 10% (2/19) of embryos double heterozygous for *Gbx2* in the pharyngeal endoderm (i.e., *Gbx2^+/flox^;Pax9Cre*) had an abnormal 4th PAA (Table 2), there was a significant increase in the penetrance of 4th PAA defects (7/14; *p* < 0.05) in the embryos null for *Gbx2* in the pharyngeal endoderm (i.e., *Gbx2^−/flox^;Pax9Cre*) (Figure 7D; Table 2). Examining *Gbx2^−/flox^;Pax9Cre* mutant mice at E15.5 by MRI revealed cardiovascular defects including RAA, A-RSA and absent common carotid arteries in 3/14 embryos analysed (Figure 7F; Table 1). Despite a relatively low incidence of cardiovascular defects in embryos with a conditional *Pax9Cre* driven deletion of *Gbx2*, this data collectively demonstrates that a genetic interaction between *Gbx2* and *Pax9* is taking place in the pharyngeal endoderm.

### 3.5. Exploring a Genetic Interaction between Gbx2, Pax9 and Tbx1

**Figure 8:**
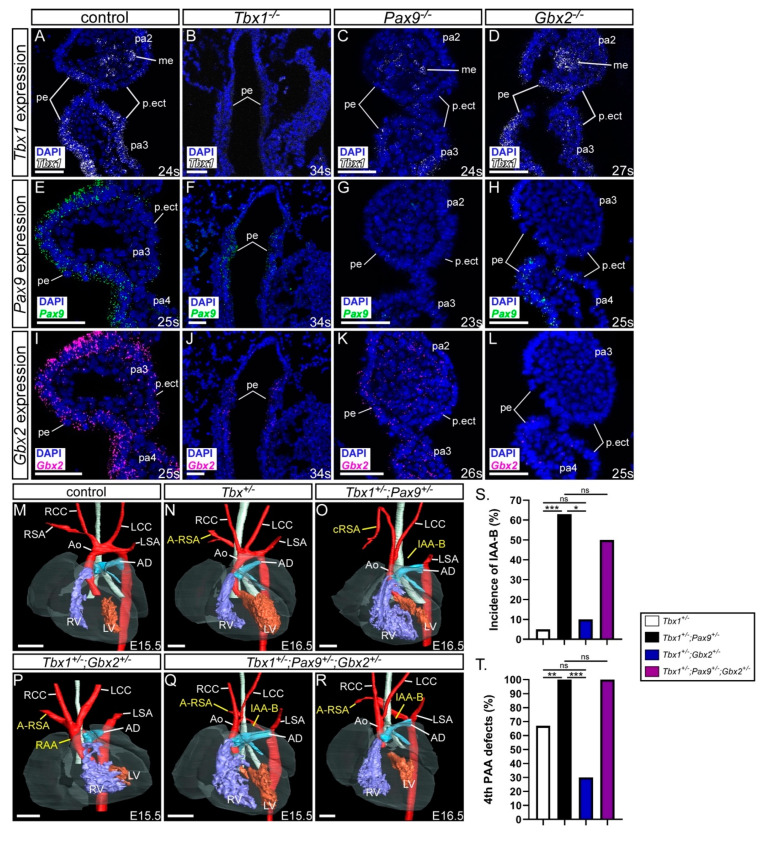
*Gbx2* heterozygosity does not modify the *Tbx1;Pax9* double heterozygous phenotype. (**A**–**L**) *Tbx1*, *Pax9* and *Gbx2* expression in E9.5–E10.5 control and *Tbx1^−/−^*, *Pax9^−/−^* and *Gbx2^−/−^* embryos (n ≥ 3 embryos per probe; 23–34 somites). (**A**) *Tbx1* is expressed in the pharyngeal endoderm, mesoderm and ectoderm in control embryos. *Tbx1* expression is absent in *Tbx1^−/−^* embryos (**B**), reduced in *Pax9^−/−^* embryos (**C**), and normal in *Gbx2^−/−^* embryos (**D**). (**E**) *Pax9* is expressed in the pharyngeal endoderm in control embryos. *Pax9* expression is reduced in *Tbx1^−/−^* embryos (**F**), absent in *Pax9^−/−^* embryos (**G**), and present in *Gbx2^−/−^* embryos (**H**). (**I**) *Gbx2* is expressed in the pharyngeal endoderm in control embryos. *Gbx2* expression is reduced in *Tbx1^−/−^* embryos (**J**), reduced in *Pax9^−/−^* embryos (**K**), and absent in *Gbx2^−/−^* embryos (**L**). (**M**–**R**) 3D reconstructions of E15.5–16.5 hearts from µCT datasets. (**M**) Control embryo with normal heart and aortic arch arteries. (**N**) *Tbx1^+/−^* embryo with aberrant right subclavian artery (A-RSA). (**O**) *Tbx1^+/−^*;*Pax9^+/−^* embryo with interrupted aortic arch type B (IAA-B) and cervical origin of the right subclavian artery (cRSA). (**P**) *Tbx1^+/−^*;*Gbx2^+/−^* embryo with a right-sided aortic arch (RAA) and A-RSA. (**Q**,**R**) *Tbx1^+/−^*;*Pax9^+/−+/−^*;*Gbx2^+/−^* embryos with IAA-B and A-RSA. (**S,T**) Graphical representation of IAA-B incidence (**S**) and 4th pharyngeal arch artery (PAA) incidence (**T**) in *Tbx1;Pax9;Gbx2* embryos. * *p* < 0.05; ** *p* < 0.001; *** *p* < 0.0001; Pearson chi-squared test for associations with continuity correction. Scale bars: 500 μm in M-R; 50 μm in (**A**–**L**). Abbreviations: Ao, aorta; AD, arterial duct; LCC, left common carotid artery; LSA, left subclavian artery; LV, left ventricle; me, mesoderm; pa, pharyngeal arch; pe, pharyngeal endoderm; p.ect, pharyngeal ectoderm; RCC, right common carotid artery; RSA, right subclavian artery; RV, right ventricle; s, somite. The somite numbers given in the legend reflect the range analysed for the whole study. The figure contains representative images only.

*Tbx1* has been shown to genetically interact with *Gbx2* and *Pax9* in the pharyngeal ectoderm and endoderm respectively [25,28]. Our and published data [42] also show that *Pax9* and *Tbx1* occupy the same conserved region in the *Gbx2* locus (Figure 1) and *Tbx1*, *Pax9* and *Gbx2* are co-expressed in the pharyngeal endoderm at E9.5 and E10.5 (Figure 1C–F) [25,28]. *Pax9* and *Gbx2*, and *Tbx1* and *Gbx2*, are downregulated in the pharyngeal endoderm of *Tbx1^−/−^* and *Pax9^−/−^* mice respectively (Figure 8A–L) [24,25] but *Pax9* and *Tbx1* expression were maintained in the pharyngeal endoderm of *Gbx2^−/−^* embryos at E9.5 (Figure 8D,H). We nevertheless speculated that a gene regulatory network may be occurring in the pharyngeal endoderm involving *Gbx2*, *Pax9* and *Tbx1*. To investigate this we analysed *Gbx2^+/−^;Pax9^+/−^;Tbx1^+/−^* triple heterozygous embryos at E15.5-E16.5 by µCT imaging and compared the observed cardiovascular defects with double heterozygous *Tbx1^+/−^*;*Pax9^+/−^* and *Tbx1^+/−^*;*Gbx2^+/−^* embryos (Figure 8M–R; Table 3). Although a non-significant reduction in the penetrance of 4th PAA derived defects was seen in *Tbx1^+/−^*;*Gbx2^+/−^* embryos compared to *Tbx1^+/−^* embryos, a similar number and type of cardiovascular defect was observed between *Tbx1^+/−^*;*Pax9^+/−^* double heterozygous and *Tbx1^+/−^*;*Gbx2^+/−^;Pax9^+/−^* triple heterozygous embryos (Figure 8S,T). This suggests that there was no change to the *Tbx1^+/−^*;*Pax9^+/−^* 4th PAA-derived phenotype caused by haploinsufficiency of *Gbx2*.

## 4. Discussion

In this study, we have employed imaging and gene interaction methodologies to provide an in-depth analysis of the *Gbx2* mutant cardiovascular phenotype throughout embryogenesis and demonstrate a genetic interaction with *Pax9* in the pharyngeal endoderm.

### 4.1. Gbx2-Null Mice

Our analysis of the *Gbx2*-null cardiovascular phenotype revealed differences from two previously published papers. At mid-embryogenesis we found 71% of mutants had an abnormal 4th PAA, compared to 50% previously reported [28], and at foetal stages, we identified 64% of mutants had a cardiovascular defect, although none displayed IAA, compared to 39% previously reported where IAA was included [29]. One explanation for the cardiovascular phenotype discrepancy between studies is the *Gbx2*-null allele used. In the previously published studies, the neomycin selection cassette was retained in the first intron of *Gbx2* [32,41], whereas it was removed from the allele used in our study. It is possible that the presence or absence of the neomycin cassette, which has been described to alter mutant mouse phenotypes [45], may have influenced the presentation of the cardiovascular phenotype. Alternatively, some cardiovascular defects may have been overlooked in the previous reports, or a subtle change in genetic background may influence the presentation of phenotypes.

The cardiovascular structures predominantly affected in *Gbx2*-null embryos were the OFT, with DORV seen in 40% of mutants, and the 4th PAA-derived vessels, with 50% of mutants having an aberrant subclavian artery which was either retro-oesphageal or isolated. Although the formation of the 4th PAA was more frequently observed to be affected at mid-embryogenesis in *Gbx2*-null embryos, with this vessel seen to be absent or hypoplastic in 71% of mutants, this did not translate into an IAA-B phenotype at the foetal stage as would be expected if the left 4th PAA had failed to form. Immunolabelling of *Gbx2^−/−^* embryos showed a disorganisation of the endothelial cells within the 4th pharyngeal arch indicating that the 4th PAA fails to lumenise. This has also been previously described in *Gbx2^−/−^* embryos [29], as well as in other mouse models where the failure of the 4th PAA is a feature, for example, in *Pax9^−/−^* [25] and *Tbx1* mutant [28] embryos. The discrepancy in 4th PAA defects observed at an earlier developmental stage, yet not manifesting into a defect at a later stage, is a well-known phenomenon observed in *Tbx1^+/−^* embryos [25,28,46,47,48,49]. The recovery of the left 4th PAA may explain why we did not observe IAA-B in the *Gbx2*-null embryos.

Patients that present with an incomplete reversal of the internal organs are diagnosed with heterotaxy which refers to any defect of left-right patterning and arrangement of the visceral organs. We identified previously unreported left-right patterning defects in a third of all *Gbx2*-null mutants examined. We did not find any change in *Pitx2c* expression in the left lateral plate mesoderm in *Gbx2*-null embryos at E8.5, although we did not pre-select embryos for in situ hybridisation analysis based on any morphological abnormalities such as the orientation of the OFT. Gbx2 may function in left-right patterning independently of Pitx2c. Left-right patterning defects are largely a result of abnormalities at the node of the primitive streak from E7.0 [50] and *Gbx2* is expressed in all three germ layers at gastrulation in the posterior epiblast [30], a region encompassing the node. Overall, the number of *Gbx2^−/−^* embryos obtained was significantly lower than expected showing that more than half of *Gbx2^−/−^* embryos are lost prior to E8.5 in early embryogenesis, regardless of the accompanying *Pax9* genotype. Early *Gbx2* expression could, therefore, be critical for gastrulation and some embryos lacking *Gbx2* may not proceed through development at this stage. The surviving embryos may then go on to develop left-right patterning and cardiovascular defects. Alternatively, *Gbx2* may function downstream of *Pitx2c* to regulate the asymmetric development of the internal organs. *Pitx2* is a regulator of gene expression [51] and the *Pitx2c* isoform plays an important role in the final stages of organ asymmetry [52] including aortic arch and outflow tract development [53,54]. *Pitx2c* mutant mice have OFT defects due to abnormal proliferation within the OFT myocardium [55]. *Gbx2* may, therefore, be a downstream target of *Pitx2c* and further work is required to investigate this possibility.

### 4.2. Genetic Interaction with Pax9

Mice double heterozygous for *Gbx2* and *Pax9* were under-represented at weaning, and some were found to die at birth with cardiovascular defects. These data indicate there is a genetic interaction between *Pax9* and *Gbx2* in cardiovascular development. Interestingly, the RAA and IAA-B phenotypes observed in *Gbx2^+/−^;Pax9^+/−^* neonates are commonly seen in *Gbx2^−/−^* and *Pax9^−/−^* mice respectively [25,29] but not in single heterozygous mice for these genes. When *Gbx2^−/−^* mice were also heterozygous for *Pax9* (i.e., *Gbx2^−/−^*;*Pax9^+/−^*), 100% of embryos and neonates presented with a cardiovascular defect (significantly increased when compared to 64% of *Gbx2^−/−^* mice), including left-right patterning defects, and there was a significantly increased incidence of IAA-B. Moreover, CAT was observed, a phenotype not previously identified in *Gbx2^−/−^* or *Pax9^−/−^* mice [25,29]. When we produced embryos null for *Pax9* and heterozygous for *Gbx2* (i.e., *Gbx2^+/−^*;*Pax9^−/−^*), or double knockouts, they all presented with typical *Pax9*-null defects but also additionally displayed CAT or AVSD. Embryos with a *Pax9^−/−^* genotype, however, did not display a left-right patterning defect as seen in the *Gbx2^−/−^* mice. Our data suggest that a reduction in bi-allelic *Gbx2* and *Pax9* expression leads to a modification of the phenotypes typically seen in the single-gene knockout mice.

The cardiovascular defects observed in the *Gbx2;Pax9* mutant mice such as AVSD, CAT and DORV are reminiscent of abnormal SHF development. Two-thirds of patients with heterotaxy have AVSD [56] and this defect arises from the failure of the SHF-derived dorsal mesenchymal protrusion to complete atrioventricular septation [57]. Patients with 22q11DS, where hemizygous expression of *TBX1* underlies the observed cardiovascular defects, may present with CAT [58], and this phenotype is fully penetrant in *Tbx1*-null mice [34,59]. *Tbx1* is expressed in the SHF [60], which overlaps with the pharyngeal endoderm [61], where *Pax9* and *Gbx2* are also expressed. It is therefore feasible that the pharyngeal endoderm expression of *Pax9* and *Gbx2* are influencing the formation of SHF-derived cardiovascular structures. There could also be a defective contribution in the *Gbx2;Pax9* mutant mice from neural crest cells (NCC) migrating through the pharyngeal arches and into the OFT where they participate in septation [8]. Mouse models with NCC defects often present with CAT [62,63]. It has been reported that a reduced number of NCC are present within the caudal pharyngeal arches in *Pax9*-null embryos at E10.5 [25] and *Gbx2*-null mice show aberrant migration of NCC [28,29]. A NCC defect, combined with deficiencies in the pharyngeal endoderm component of the SHF, could, therefore, become more penetrant on a *Gbx2;Pax9* mutant background resulting in a higher incidence of the CAT and IAA-B phenotypes [64].

### 4.3. Gbx2 and Pax9 Interact in the Pharyngeal Endoderm

Cardiovascular defects were observed when *Gbx2* was conditionally deleted from the developing embryo using the *Pax9Cre* allele, and these were at a comparable level to that observed in the constitutive double heterozygous embryos, although the IAA-B phenotype was not seen. A higher incidence of 4th PAA defects, however, was seen in the conditional mutants at E10.5 when analysed by ink injection compared to those observed at foetal stages. As discussed above, it is well recognised that the 4th PAA can recover during development and there is not always a direct correlation to defects that are subsequently seen at the foetal stages. At the E10.5 stage, half the conditional *Gbx2^−/flox^;Pax9Cre* embryos presented with 4th PAA defects, whereas 100% of the constitutive *Gbx2^−/−^*;*Pax9^+/−^* embryos did, suggesting that either 4th PAA morphogenesis is only partly controlled from the pharyngeal endoderm or the Cre-mediated recombination of the *Gbx2*-floxed allele is not 100% efficient. Residual expression of *Gbx2* RNA was observed in the pharyngeal endoderm of *Gbx2^−/flox^;Pax9Cre* embryos (Figure 7B), which may be due to the concomitant onset of *Pax9Cre* and *Gbx2* expression at E8.5 resulting in a slight delay in Cre activity on the *Gbx2*-floxed allele. It has previously been shown that the deletion of *Gbx2* from the pharyngeal ectoderm using *AP2αCre* mice resulted in a 47% penetrance of 4th PAA defects at E10.5 with the distribution of unilateral and bilateral 4th PAA defects in conditional endoderm and ectoderm mutants being very similar [28]. It is therefore tempting to speculate that *Gbx2* expression from both pharyngeal epithelial tissues is required for correct 4th PAA morphogenesis. The incidence of defects seen at foetal and embryonic stages in the constitutive *Gbx2^−/−^*, *Pax9^−/−^* and *Gbx2;Pax9* complex mutants was fairly concordant indicating that 4th PAA defects observed at E10.5 in these genotypes did proceed directly to arch artery defects at foetal stages.

### 4.4. The Tbx1;Pax9 Double Heterozygous Phenotype is not Modulated by Gbx2 Haploinsufficiency

Mice heterozygous for *Tbx1* typically have defects affecting the 4th PAA such as IAA and A-RSA [65], but have also been shown to present with further cardiac defects such as VSD [59,66]. *Tbx1* has been shown to genetically interact with *Pax9* and *Gbx2* in the pharyngeal endoderm and ectoderm, respectively [25,28]. We identified three PAX binding sites in a highly conserved region of the *GBX2* locus, as well as a previously validated TBX binding site [42]. In luciferase assays, PAX9 was able to activate the *Gbx2* conserved region although TBX1 did not, and when PAX9 and TBX1 were co-expressed, PAX9 was not able to activate the reporter. This suggests that PAX9 can activate Gbx2 through binding to the conserved region, whereas TBX1 acts as a repressor. However, to fully provide conclusive evidence for a molecular interaction for *Gbx2* expression to be driven by Pax9 or Tbx1, further experiments would need to be performed such as an electromobility shift assay or chromatin immunoprecipitation experiments. Mutating the putative binding sites in the luciferase assay would also determine specificity. Gene expression is highly dynamic during cardiovascular development with constant switching between gene activation and suppression [67]. For example, *Tbx1* acts as a poised enhancer to bind and keep DNA in the open state to then recruit other transcription or regulatory factors. It has been shown that Tbx1 recruits P53 to *Gbx2* to modulate *Gbx2* expression through histone methylation [42]. As Tbx1 also regulates histone modification [68] it could have a potential role in controlling the timely activation of *Gbx2* by Pax9 during cardiovascular development.

*Tbx1*, *Pax9* and *Gbx2* are all expressed in the pharyngeal endoderm at E9.5 and E10.5, and could, therefore, function together in the endoderm which is acting as a signalling centre to interact with other tissue types required in PAA morphogenesis. *Pax9* and *Tbx1* were each downregulated in *Tbx1^−/−^* and *Pax9^−/−^* embryos respectively, which suggests that *Tbx1* and *Pax9* act in a non-hierarchal pathway [25]. *Tbx1* and *Pax9* expression in *Gbx2^−/−^* embryos, however, were unaffected indicating that these genes function hierarchically upstream of *Gbx2*. This is reflected in our phenotyping data where we did not identify any difference in the incidence of 4th PAA-derived defects at foetal stages in *Tbx1^+/−^; Pax9^+/−^;Gbx2^+/−^* triple heterozygous mutants compared to *Tbx1^+/−^; Pax9^+/−^* double heterozygous mutants. Of note, however, in our study we only identified a low penetrance of 4th PAA-derived defects in *Tbx1^+/−^;Gbx2^+/−^* mutants, and only one incidence (10%) of IAA-B. This is in contrast to that previously shown for embryos of the same genotype where 27% of mutants (6 out of 22) had IAA-B [28]. This could possibly be attributed to the use of a different *Gbx2* targeted allele as discussed above.

## 5. Conclusions

In summary, a genetic regulatory network comprising of *Tbx1*, *Pax9* and *Gbx2* is required to control morphogenesis of the arch arteries and the outflow tract of the developing cardiovascular system, and this interaction occurs, in part, within the pharyngeal epithelia at mid-embryogenesis in the mouse. An earlier role for *Gbx2* in establishing left-right patterning requires further investigation.

## Figures and Tables

**Table 1:** Number of cardiovascular defects observed in *Gbx2;Pax9* mutant mice.

Genotype	*n*	Abnormal	VSD	AVSD	DORV + IVC	CAT	RAA/RAD +/or A-SA *^a^*	IAA-B +/− A-RSA	Absent CC *^b^*	L-R Defect *^c^*
*Gbx2^−/−^*	25	16 (64%)	2 (8%)	0	10 (40%)	0	13 (52%)	0	0	9 (36%)
*Gbx2^+/^* *^−^;* *Pax9^+/^* *^−^*	28	6 (21%)	0	0	0	0	4 (14%)	2 (7%)	0	0
*Gbx2^−/−^; Pax9^+/−^*	14	14 (100%) *	3 (21%)	0	7 (50%)	1 (7%)	8 (57%)	5 ** (36%)	2 (16%)	6 (43%)
*Pax9^−/−^*	9	9 (100%)	2 (22%)	0	3 (44%)	0	0	8 (89%)	5 (56%)	0
*Gbx2^+/−^; Pax9^−/−^*	9	9 (100%)	2 (22%)	0	5 (56%)	2 (22%)	1 (11%)	8 (89%)	6 (67%)	0
*Gbx2^−/−^; Pax9^−/−^*	2	2 (100%)	0	1 (50%)	0	1 (50%)	1 (50%)	1 (50%)	1 (50%)	0
*Gbx2^−/flox^;* *Pax9Cre*	14	3 (21%)	0	0	0	0	3 (21%)	0	1 (7%)	0

Embryos and neonates were collected from *Gbx2^+/^^−^;Pax9^+/^^−^* and *Gbx2^flox^;Pax9Cre* crosses. All embryos were analysed at E13.5-E15.5 by MRI, µCT and histology. Neonates were analysed by dissection at P0: *Gbx2^+/^^−^;Pax9^+/^^−^* (n = 12), *Gbx2^−/−^; Pax9^+/−^* (n = 1), *Pax9^−/−^* (n = 2), *Gbx2^+/−^;Pax9^−/−^* (n = 2), and *Gbx2^−/−^;Pax9^−/−^* (n = 1). Results for embryos and neonates are combined. The outflow tract phenotype was not assessed in neonates. All wild-type (n = 12), *Pax9^+/−^* (n = 8), *Gbx2^+/−^* (n = 22), *Gbx2^+/flox^* (n = 6), *Gbx2^−/flox^* (n = 9) and *Pax9Cre* (n = 12) control embryos and neonates assessed were normal. There was a statistically significant increase in the incidence of cardiovascular defects in total, and IAA-B specifically, in *Gbx2^−/−^;Pax9^+/−^* embryos compared to the *Gbx2^−/−^* embryos. * *p* < 0.05, ** *p* = 0.01 (Fisher’s exact test). *^a^* A-SA refers to a right or left retro-esophageal subclavian artery, cervical origin of the RSA, or an isolated RSA. One *Pax9^−/−^*, three *Gbx2^−/−^* and both *Gbx2^−/−^; Pax9^−/−^* embryos had isolated RSA. *^b^* Absent common carotid artery (CC), resulting in the internal and external carotid arteries arising directly from the main aortic vessels, unilaterally or bilaterally. *^c^* L-R defect refers to an overt left-right patterning defect where embryos show either right pulmonary isomerism, right atrial isomerism or mirror image branching of the aortic arch arteries. Abbreviations: AoA, aortic arch; A-RSA, aberrant right subclavian artery; A-SA, aberrant subclavian artery (left or right); AVSD, atrioventricular septal defect; DORV + IVC, double outlet right ventricle with interventricular communication; IAA-B, interruption of the aortic arch, type B; RAA, right-sided aortic arch; RAD, right-sided arterial duct; RSA, right subclavian artery; VSD, perimembranous ventricular septal defect.

**Table 2:** Number of pharyngeal arch artery defects observed in *Gbx2;Pax9* mutant embryos.

						Bilateral Defects		
Genotype	n	PAA	Abnormal (%)	Unilateral Defect	Bilateral Defect	Present	Hypo/Int/Abs	Absent
*Gbx2^+/−^*	44	4	3 (7%)	3 (7%)	0	-	-	-
*Gbx2^+/−^;* *Pax9^+/−^*	63	4	20 (32%)	13 (21%)	7 (11%)	-	3	4
*Gbx2^−/−^*	17	4	12 (71%)	7 (41%)	5 (29%)	-	3	2
*Gbx2^−/−^; Pax9^+/−^*	7	4	7 (100%)	1 (14%)	6 (86%) *	-	2	4
*Pax9^−/− a^*	22	1	13 (59%)	1 (5%)	12 (55%)	11	1	-
		2	8 (36%)	3 (14%)	5 (23%)	4	1	-
		3	17 (77%)	3 (14%)	14 (64%)	-	10	4
		4	22 (100%)	1 (5%)	21 (95%)	-	3	18
*Gbx2^+/−^;* *Pax9^−/−^*	6	1	6 (100%)	0	6 (100%)	6	-	-
		2	2 (33%)	2 (33%)	0	-	-	-
		3	6 (100%)	0	6 (100%)	-	1	5
		4	6 (100%)	0	6 (100%)	-	-	6
*Gbx2^−/−^;* *Pax9^−/−^*	1	4	1 (100%)	0	1 (100%)	-	-	1
*Gbx2^+/flox^;* *Pax9Cre*	19	4	2 (10%)	1 (5%)	1 (5%)	-	-	1
*Gbx2^−/flox^;* *Pax9Cre*	14	4	7 (50%) *	5 (36%)	2 (14%)	-	1	1

Embryos were collected from *Gbx2^+/^^−^;Pax9^+/^^−^* and *Gbx2^flox^;Pax9Cre* crosses and assessed for PAA defects by intracardiac ink injections. *Gbx2^−/−^* embryos were also imaged using HREM (E10.5, n = 3 and E11.5, n = 5) to visualise the PAA. Data are combined. For *Pax9^−/−^* and *Gbx2^+/^^−^;Pax9^−/−^* genotypes each left and right PAA 1–4 was scored as having a unilateral or bilateral defect, and the bilateral defects categorised as either present, a combination of hypoplastic, interrupted and/or absent (Hypo/Int/Abs), and bilaterally absent. For all other genotypes ink injection was performed at E10.5 and only the 4th PAA was scored. All control embryos, *Pax9^+/−^* (n = 18), *Gbx2^+/flox^* (n = 22) and *Gbx2^−/flox^* (n = 15) were normal. There was a significantly increased incidence of bilateral 4th PAA defects in *Gbx2^−/−^; Pax9^+/−^* embryos compared to *Gbx2^−/−^* embryos, and abnormal 4th PAA defects in *Gbx2^−/flox^; Pax9Cre* embryos compared to *Gbx2^+/flox^; Pax9Cre* embryos. * *p* < 0.05 (Pearson’s Chi-square test for associations). *^a^* New data (n = 2) combined with published data (n = 20) [25].

**Table 3:** Number of cardiovascular defects observed in *Tbx1;Gbx2;Pax9* mutant mice.

Genotype	*n*	VSD	RAA +/or A-RSA	IAA-B +/− A-RSA	4th PAA Defect
*Tbx1^+/− a^*	21	2 (10%)	13 (62%)	1 (5%)	14 (67%)
*Tbx1^+/^* *^−^;* *Pax9^+/^* *^− b^*	24	5 (21%)	9 (38%)	15 (63%) **	24 (100%)
*Tbx1^+/−^; Gbx2^+/−^*	10	3(30%)	2(20%)	1(10%)	3(30%)
*Tbx1^+/−^; Gbx2^+/−^; Pax9^+/−^*	8	3 (38%)	4 (50%)	4 (50%)	8 (100%)

Embryos were collected from *Tbx1^+/^^−^;Gbx2^+/^^−^* and *Pax9^+/^^−^* crosses. Embryos were analysed by µCT at E13.5-E16.5. There was a statistically significant increase in the incidence of IAA-B in the *Tbx1^+/−^;Pax9^+/−^* embryos compared with the *Tbx1^+/−^* embryos. ** *p* < 0.0001 (Pearson’s chi-squared test for associations with continuity correction). *^a^* New data (n = 3) combined with published MRI data (n = 19) [25]. *^b^* New data (n = 4) combined with published MRI data (n = 20) [25]. Abbreviations: A-RSA, aberrant right subclavian artery; IAA-B, interruption of the aortic arch, type B; RAA, right-sided aortic arch; VSD, perimembranous ventricular septal defect.

## Supplementary Materials

The following are available online at https://www.mdpi.com/2308-3425/7/2/20/s1. Figure S1. No *Gbx2* mRNA is detected in *Gbx2^−/−^* embryos created by recombining the *Gbx2^flox^* allele with *Sox2Cre* at E9.5; Figure S2. Thymus and palate abnormalities seen in *Gbx2;Pax9* mutant embryos and neonates; Figure S3. *Pax9Cre* activity in the pharyngeal endoderm in E9.5 embryos. Table S1. Expected and observed genotypes of embryos and foetuses collected from a *Gbx2^+/−^* intercross; Table S2. Expected and observed genotypes of weaned pups from a *Gbx2^+/−^* × *Pax9^+/−^* cross; Table S3. Expected and observed genotypes of embryos and foetuses collected from a *Gbx2^+/−^;Pax9^+/−^* intercross; Table S4. Summary of thymus phenotypes observed in *Gbx2* and *Gbx2;Pax9* mutant embryos at E15.5 and neonates at P0; Table S5. Antibodies and probes used for immunostaining and in situ hybridisation.

## References

[1] Andersen T.A., Troelsen Kde L., Larsen L.A. (2014). Of mice and men: Molecular genetics of congenital heart disease. Cell. Mol. Life Sci..

[2] Boudjemline Y., Fermont L., Le Bidois J., Lyonnet S., Sidi D., Bonnet D. (2001). Prevalence of 22q11 deletion in fetuses with conotruncal cardiac defects: A 6-year prospective study. J. Pediatr..

[3] Papangeli I., Scambler P. (2013). The 22q11 deletion: DiGeorge and velocardiofacial syndromes and the role of TBX1. Wiley Interdiscip. Rev. Dev. Biol..

[4] Yagi H., Furutani Y., Hamada H., Sasaki T., Asakawa S., Minoshima S., Ichida F., Joo K., Kimura M., Imamura S. (2003). Role of TBX1 in human del22q11.2 syndrome. Lancet.

[5] Zweier C., Sticht H., Aydin-Yaylagul I., Campbell C.E., Rauch A. (2007). Human TBX1 missense mutations cause gain of function resulting in the same phenotype as 22q11.2 deletions. Am. J. Hum. Genet..

[6] Kelly R.G. (2012). The second heart field. Curr. Top. Dev. Biol..

[7] Anderson R.H., Moorman A.F., Brown N.A., Bamforth S.D., Chaudhry B., Henderson D.J., Mohun T.J., da Cruz E., Ivy D., Jaggers J. (2014). Normal and abnormal development of the heart. Pediatric and Congenital Cardiology, Cardiac Surgery and Intensive Care.

[8] Waldo K., Miyagawa-Tomita S., Kumiski D., Kirby M.L. (1998). Cardiac neural crest cells provide new insight into septation of the cardiac outflow tract: Aortic sac to ventricular septal closure. Dev. Biol..

[9] Bajolle F., Zaffran S., Kelly R.G., Hadchouel J., Bonnet D., Brown N.A., Buckingham M.E. (2006). Rotation of the myocardial wall of the outflow tract is implicated in the normal positioning of the great arteries. Circ. Res..

[10] Anderson R.H., Wessels A., Vettukattil J.J. (2010). Morphology and Morphogenesis of Atrioventricular Septal Defect With Common Atrioventricular Junction. World J. Pediatr. Congenit. Heart Surg..

[11] Wang X., Chen D., Chen K., Jubran A., Ramirez A., Astrof S. (2017). Endothelium in the pharyngeal arches 3, 4 and 6 is derived from the second heart field. Dev. Biol..

[12] Graham A., Smith A. (2001). Patterning the pharyngeal arches. Bioessays.

[13] Chapman D.L., Garvey N., Hancock S., Alexiou M., Agulnik S.I., Gibson-Brown J.J., Cebra-Thomas J., Bollag R.J., Silver L.M., Papaioannou V.E. (1996). Expression of the T-box family genes,Tbx1-Tbx5, during early mouse development. Dev. Dyn..

[14] Veitch E., Begbie J., Schilling T.F., Smith M.M., Graham A. (1999). Pharyngeal arch patterning in the absence of neural crest. Curr. Biol..

[15] Piotrowski T., Nusslein-Volhard C. (2000). The endoderm plays an important role in patterning the segmented pharyngeal region in zebrafish (Danio rerio). Dev. Biol..

[16] McCauley D.W., Bronner-Fraser M. (2003). Neural crest contributions to the lamprey head. Development.

[17] Graham A., Okabe M., Quinlan R. (2005). The role of the endoderm in the development and evolution of the pharyngeal arches. J. Anat..

[18] Edlund R.K., Ohyama T., Kantarci H., Riley B.B., Groves A.K. (2014). Foxi transcription factors promote pharyngeal arch development by regulating formation of FGF signaling centers. Dev. Biol..

[19] Hasten E., Morrow B.E. (2019). Tbx1 and Foxi3 genetically interact in the pharyngeal pouch endoderm in a mouse model for 22q11.2 deletion syndrome. PLoS Genet..

[20] Jackson A., Kasah S., Mansour S.L., Morrow B., Basson M.A. (2014). Endoderm-specific deletion of Tbx1 reveals an FGF-independent role for Tbx1 in pharyngeal apparatus morphogenesis. Dev. Dyn..

[21] Hiruma T., Nakajima Y., Nakamura H. (2002). Development of pharyngeal arch arteries in early mouse embryo. J. Anat..

[22] Bamforth S.D., Chaudhry B., Bennett M., Wilson R., Mohun T.J., Van Mierop L.H., Henderson D.J., Anderson R.H. (2013). Clarification of the identity of the mammalian fifth pharyngeal arch artery. Clin. Anat..

[23] Suntratonpipat S., Bamforth S.D., Johnson A.L., Noga M., Anderson R.H., Smallhorn J., Tham E. (2015). Childhood presentation of interrupted aortic arch with persistent carotid ducts. World J. Pediatr. Congenit. Heart Surg..

[24] Ivins S., van Lammerts Beuren K., Roberts C., James C., Lindsay E., Baldini A., Ataliotis P., Scambler P.J. (2005). Microarray analysis detects differentially expressed genes in the pharyngeal region of mice lacking Tbx1. Dev. Biol..

[25] Phillips H.M., Stothard C.A., Shaikh Qureshi W.M., Kousa A.I., Briones-Leon J.A., Khasawneh R.R., O’Loughlin C., Sanders R., Mazzotta S., Dodds R. (2019). Pax9 is required for cardiovascular development and interacts with Tbx1 in the pharyngeal endoderm to control 4th pharyngeal arch artery morphogenesis. Development.

[26] Neubuser A., Koseki H., Balling R. (1995). Characterization and developmental expression of Pax9, a paired-box-containing gene related to Pax1. Dev. Biol..

[27] Peters H., Neubuser A., Kratochwil K., Balling R. (1998). Pax9-deficient mice lack pharyngeal pouch derivatives and teeth and exhibit craniofacial and limb abnormalities. Genes Dev..

[28] Calmont A., Ivins S., Van Bueren K.L., Papangeli I., Kyriakopoulou V., Andrews W.D., Martin J.F., Moon A.M., Illingworth E.A., Basson M.A. (2009). Tbx1 controls cardiac neural crest cell migration during arch artery development by regulating Gbx2 expression in the pharyngeal ectoderm. Development.

[29] Byrd N.A., Meyers E.N. (2005). Loss of Gbx2 results in neural crest cell patterning and pharyngeal arch artery defects in the mouse embryo. Dev. Biol..

[30] Li J.Y.H., Joyner A.L. (2001). *Otx2* and *Gbx2* are required for refinement and not induction of mid-hindbrain gene expression. Development.

[31] Bouillet P., Chazaud C., Oulad-Abdelghani M., Dollé P., Chambon P. (1995). Sequence and expression pattern of the Stra7 (Gbx-2) homeobox-containing gene induced by retinoic acid in P19 embryonal carcinoma cells. Dev. Dyn..

[32] Li J.Y., Lao Z., Joyner A.L. (2002). Changing requirements for Gbx2 in development of the cerebellum and maintenance of the mid/hindbrain organizer. Neuron.

[33] Hayashi S., Lewis P., Pevny L., McMahon A.P. (2002). Efficient gene modulation in mouse epiblast using a Sox2Cre transgenic mouse strain. Mech. Dev..

[34] Jerome L.A., Papaioannou V.E. (2001). DiGeorge syndrome phenotype in mice mutant for the T-box gene, Tbx1. Nat. Genet..

[35] Srinivas S., Watanabe T., Lin C.S., William C.M., Tanabe Y., Jessell T.M., Costantini F. (2001). Cre reporter strains produced by targeted insertion of EYFP and ECFP into the ROSA26 locus. BMC Dev. Biol..

[36] Geyer S.H., Mohun T.J., Weninger W.J. (2009). Visualizing Vertebrate Embryos with Episcopic 3D Imaging Techniques. Sci. World J..

[37] Degenhardt K., Wright A.C., Horng D., Padmanabhan A., Epstein J.A. (2010). Rapid 3D phenotyping of cardiovascular development in mouse embryos by micro-CT with iodine staining. Circ. Cardiovasc. Imaging.

[38] Bamforth S.D., Schneider J.E., Bhattacharya S. (2012). High-throughput analysis of mouse embryos by magnetic resonance imaging. Cold Spring Harb. Protoc..

[39] Schneider J.E., Bose J., Bamforth S.D., Gruber A.D., Broadbent C., Clarke K., Neubauer S., Lengeling A., Bhattacharya S. (2004). Identification of cardiac malformations in mice lacking Ptdsr using a novel high-throughput magnetic resonance imaging technique. BMC Dev. Biol..

[40] Ogawa T., Kapadia H., Feng J.Q., Raghow R., Peters H., D’Souza R.N. (2006). Functional Consequences of Interactions between Pax9 and Msx1 Genes in Normal and Abnormal Tooth Development. J. Biol. Chem..

[41] Wassarman K.M., Lewandoski M., Campbell K., Joyner A.L., Rubenstein J.L., Martinez S., Martin G.R. (1997). Specification of the anterior hindbrain and establishment of a normal mid/hindbrain organizer is dependent on Gbx2 gene function. Development.

[42] Caprio C., Baldini A. (2014). p53 suppression partially rescues the mutant phenotype in mouse models of DiGeorge syndrome. Proc. Natl. Acad. Sci. USA.

[43] Brennan J., Norris D.P., Robertson E.J. (2002). Nodal activity in the node governs left-right asymmetry. Genes Dev..

[44] Van Praagh R., Van Praagh S. (1965). The anatomy of common aorticopulmonary trunk (truncus arteriosus communis) and its embryologic implications. A study of 57 necropsy cases. Am. J. Cardiol..

[45] Olson E.N., Arnold H.H., Rigby P.W.J., Wold B.J. (1996). Know Your Neighbors: Three Phenotypes in Null Mutants of the Myogenic bHLH Gene MRF4. Cell.

[46] Lindsay E.A., Baldini A. (2001). Recovery from arterial growth delay reduces penetrance of cardiovascular defects in mice deleted for the DiGeorge syndrome region. Hum. Mol. Genet..

[47] Guris D.L., Duester G., Papaioannou V.E., Imamoto A. (2006). Dose-dependent interaction of Tbx1 and Crkl and locally aberrant RA signaling in a model of del22q11 syndrome. Dev. Cell..

[48] Randall V., McCue K., Roberts C., Kyriakopoulou V., Beddow S., Barrett A.N., Vitelli F., Prescott K., Shaw-Smith C., Devriendt K. (2009). Great vessel development requires biallelic expression of Chd7 and Tbx1 in pharyngeal ectoderm in mice. J. Clin. Investig..

[49] Ryckebusch L., Bertrand N., Mesbah K., Bajolle F., Niederreither K., Kelly R.G., Zaffran S. (2010). Decreased Levels of Embryonic Retinoic Acid Synthesis Accelerate Recovery From Arterial Growth Delay in a Mouse Model of DiGeorge Syndrome. Circ. Res..

[50] Shiratori H., Hamada H. (2006). The left-right axis in the mouse: From origin to morphology. Development.

[51] Cox C.J., Espinoza H.M., McWilliams B., Chappell K., Morton L., Hjalt T.A., Semina E.V., Amendt B.A. (2002). Differential regulation of gene expression by PITX2 isoforms. J. Biol. Chem..

[52] Liu C., Liu W., Lu M.F., Brown N.A., Martin J.F. (2001). Regulation of left-right asymmetry by thresholds of Pitx2c activity. Development.

[53] Franco D., Campione M. (2003). The role of Pitx2 during cardiac development. Linking left-right signaling and congenital heart diseases. Trends Cardiovasc. Med..

[54] Liu C., Liu W., Palie J., Lu M.F., Brown N.A., Martin J.F. (2002). Pitx2c patterns anterior myocardium and aortic arch vessels and is required for local cell movement into atrioventricular cushions. Development.

[55] Ai D., Liu W., Ma L., Dong F., Lu M.F., Wang D., Verzi M.P., Cai C., Gage P.J., Evans S. (2006). Pitx2 regulates cardiac left-right asymmetry by patterning second cardiac lineage-derived myocardium. Dev. Biol..

[56] Burns T., Yang Y., Hiriart E., Wessels A. (2016). The Dorsal Mesenchymal Protrusion and the Pathogenesis of Atrioventricular Septal Defects. J. Cardiovasc. Dev. Dis..

[57] Snarr B.S., O’Neal J.L., Chintalapudi M.R., Wirrig E.E., Phelps A.L., Kubalak S.W., Wessels A. (2007). Isl1 expression at the venous pole identifies a novel role for the second heart field in cardiac development. Circ. Res..

[58] Unolt M., Versacci P., Anaclerio S., Lambiase C., Calcagni G., Trezzi M., Carotti A., Crowley T.B., Zackai E.H., Goldmuntz E. (2018). Congenital heart diseases and cardiovascular abnormalities in 22q11.2 deletion syndrome: From well-established knowledge to new frontiers. Am. J. Med. Genet. Part A.

[59] Merscher S., Funke B., Epstein J.A., Heyer J., Puech A., Lu M.M., Xavier R.J., Demay M.B., Russell R.G., Factor S. (2001). TBX1 is responsible for cardiovascular defects in velo-cardio-facial/DiGeorge syndrome. Cell.

[60] Xu H., Morishima M., Wylie J.N., Schwartz R.J., Bruneau B.G., Lindsay E.A., Baldini A. (2004). Tbx1 has a dual role in the morphogenesis of the cardiac outflow tract. Development.

[61] Park E.J., Ogden L.A., Talbot A., Evans S., Cai C.L., Black B.L., Frank D.U., Moon A.M. (2006). Required, tissue-specific roles for Fgf8 in outflow tract formation and remodeling. Development.

[62] Conway S.J., Henderson D.J., Copp A.J. (1997). Pax3 is required for cardiac neural crest migration in the mouse: Evidence from the splotch (Sp2H) mutant. Development.

[63] Keyte A., Hutson M.R. (2012). The neural crest in cardiac congenital anomalies. Differentiation.

[64] Bradshaw L., Chaudhry B., Hildreth V., Webb S., Henderson D.J. (2009). Dual role for neural crest cells during outflow tract septation in the neural crest-deficient mutant Splotch(2H). J. Anat..

[65] Lindsay E.A., Vitelli F., Su H., Morishima M., Huynh T., Pramparo T., Jurecic V., Ogunrinu G., Sutherland H.F., Scambler P.J. (2001). Tbx1 haploinsufficieny in the DiGeorge syndrome region causes aortic arch defects in mice. Nature.

[66] Zhang Z., Baldini A. (2008). In vivo response to high-resolution variation of Tbx1 mRNA dosage. Hum. Mol. Genet..

[67] Dupays L., Mohun T. (2017). Spatiotemporal regulation of enhancers during cardiogenesis. Cell. Mol. Life Sci. CMLS.

[68] Fulcoli F.G., Franzese M., Liu X., Zhang Z., Angelini C., Baldini A. (2016). Rebalancing gene haploinsufficiency in vivo by targeting chromatin. Nat. Commun..

