# Supplementary material for: Pax9 and Gbx2 Interact in the Pharyngeal Endoderm to Control Cardiovascular Development
Source: J Cardiovasc Dev Dis. 2020 May 25;7(2):20. doi: 10.3390/jcdd7020020 (PMC7344924; doi:10.3390/jcdd7020020)
Supplement: Supplementary file 1 [file jcdd-07-00020-s001.pdf]

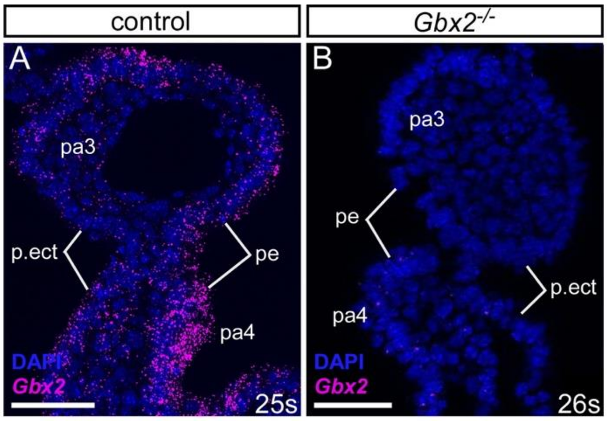

Figure S1. No *Gbx2* mRNA is detected in *Gbx2*$^{-/-}$ embryos created by recombining the *Gbx2*^{flox} allele with *Sox2Cre* at E9.5. (**A**) RNAscope in situ hybridisation shows *Gbx2* mRNA expression in the pharyngeal arch (pa) ectoderm (p.ect) and endoderm (pe) in wild-type embryos (n = 5, 24–28 somites). (**B**) No *Gbx2* mRNA is detected in *Gbx2*$^{-/-}$ embryos (n = 3, 25–27 somites). Scale bars: 50 μm. The somite numbers given in the legend reflect the range analysed for the whole study. The figure contains representative images only.

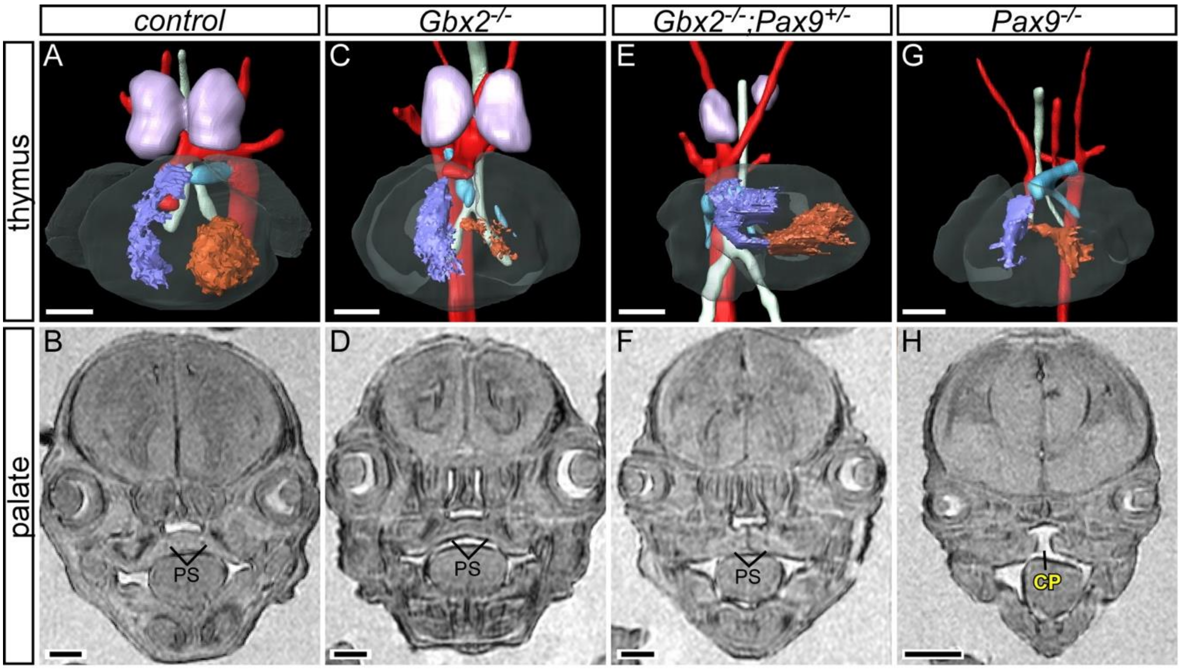

Figure S2. Thymus and palate abnormalities seen in *Gbx2;Pax9* mutant embryos and neonates. E15.5 embryos were examined by MRI. (**A**,**B**) Control embryos had normally placed thymic lobes (*purple*) located ventrally to the aortic arch arteries (**A**), and closed palatal shelves (PS; B). (**C**,**D**) Most *Gbx2*$^{-/-}$ embryos (n = 25 examined) had a normal thymus (**C**) and palate (**D**). (**E**,**F**) A large number of *Gbx2*$^{-/-}$*;Pax9*$^{+/-}$ mutants (n = 10/14 examined) had small and misplaced thymic lobes (**E**) or the thymus was absent. The palate was unaffected (**F**). (**G**,**H**) In all embryos and neonates with a *Pax9*$^{-/-}$ genotype, i.e., *Pax9*$^{-/-}$ (n = 9), *Gbx2*$^{+/-}$*;Pax9*$^{-/-}$ (n = 9), and *Gbx2*$^{-/-}$*;Pax9*$^{-/-}$ (n = 2), the thymus was absent and a cleft palate (CP) was observed. Scale, 500 μm.

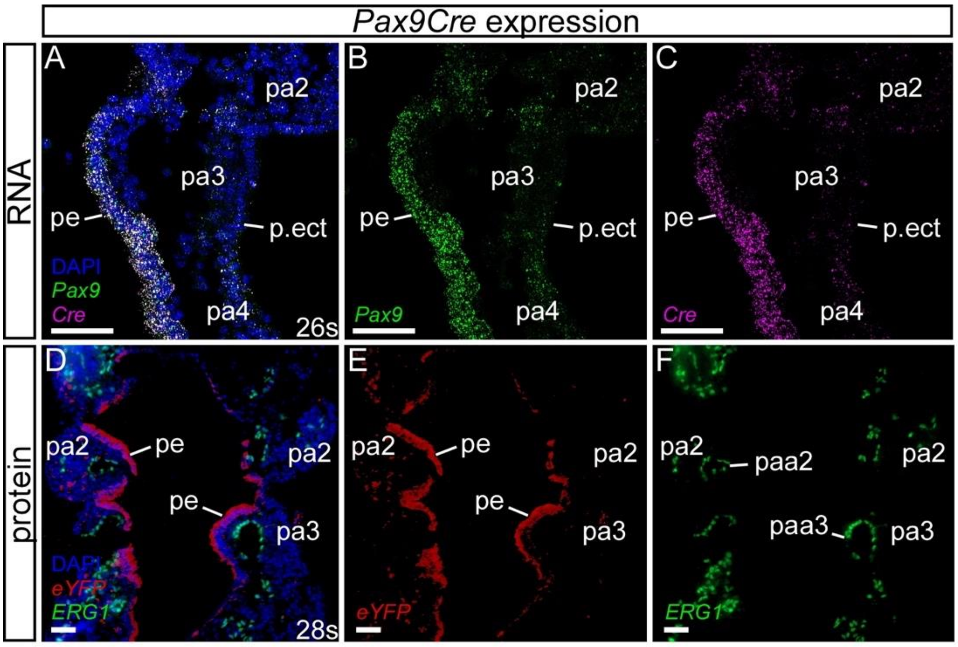

Figure S3. *Pax9Cre* activity in the pharyngeal endoderm in E9.5 embryos. (**A–C**) RNA in situ hybridisation showing *Pax9* and *Cre* expression in the pharyngeal endoderm in a *Pax9Cre* positive embryo. *Pax9* (**B**) and *Cre* expression (**C**) overlap in the pharyngeal endoderm (**A**). (**D–F**) eYFP reporter gene expression from the *Pax9Cre* allele is shown by immunostaining with an anti-eYFP antibody (red) in the pharyngeal endoderm (**D,E**). Endothelial cells have also been labelled with an anti-ERG1 antibody (**D,F**). Abbreviations: pa, pharyngeal arch; paa, pharyngeal arch artery; pe, pharyngeal endoderm; p.ect, pharyngeal ectoderm. Somite numbers are indicated (s). Scale, 50 µm.

Table S1. Expected and observed genotypes of embryos and foetuses collected from a *Gbx2*$^{+/-}$ intercross.

Genotype	Observed	Expected
Gbx2$^{+/+}$	61	67.75
Gbx2$^{+/-}$	173	135.5
Gbx2$^{-/-}$	37	67.75
Total	271	271
	Chi-square, $p = 4 \times 10^{-6}$	

Table S2. Expected and observed genotypes of weaned pups from a *Gbx2*$^{+/-}$ x *Pax9*$^{+/-}$ cross.

Genotype	Observed (3 Weeks Old)	Expected
Gbx2$^{+/+}$;*Pax9*$^{+/+}$	79	76.75
Pax9$^{+/-}$	74	76.75
Gbx2$^{+/-}$	95	76.75
Gbx2$^{+/-}$;*Pax9*$^{+/-}$	59	76.75
Total	307	307
	Chi-square, $p = 0.035$	

Table S3. Expected and observed genotypes of embryos and foetuses collected from a *Gbx2*$^{+/-}$; *Pax9*$^{+/-}$ intercross.

Genotype	Observed (E9.5-P0)	Expected
Gbx2$^{+/+}$;*Pax9*$^{+/+}$	16	10.75
Pax9$^{+/-}$	20	21.5
Gbx2$^{+/-}$	23	21.5
Pax9$^{-/-}$	9	10.75
Gbx2$^{-/-}$	5	10.75
Gbx2$^{+/-}$;*Pax9*$^{+/-}$	67	43

Gbx2$^{+/-}$;*Pax9*$^{-/-}$	19	21.5
Gbx2$^{-/-}$;*Pax9*$^{+/-}$	10	21.5
Gbx2$^{-/-}$;*Pax9*$^{-/-}$	3	10.75
Total	172	172
	Chi-square, $p = 1.12 \times 10^{-4}$	

Table S4. Summary of thymus phenotypes observed in *Gbx2* and *Gbx2;Pax9* mutant embryos at E15.5 and neonates at P0.

Genotype	Stage	n	Thymus Phenotype: Normal	Thymus Phenotype: Split/ Asymmetric/ Vestigial	Thymus Phenotype: Absent
Gbx2$^{-/-}$	E15.5	25	23 (92%)	2 (8%)	0
Gbx2$^{-/-}$;*Pax9*$^{+/-}$	E15.5	13	4 (28%)	4*** (28%)	6*** (43%)
	P0	1			
Pax9$^{-/-}$	E15.5	7	0	0	9 (100%)
	P0	2			
Gbx2$^{+/-}$;*Pax9*$^{-/-}$	E15.5	7	0	0	9 (100%)
	P0	2			
Gbx2$^{-/-}$;*Pax9*$^{-/-}$	E15.5	1	0	0	2 (100%)
	P0	1			

The thymus was significantly more frequently seen to be asymmetric in appearance and split apart, or absent, in *Gbx2*$^{-/-}$;*Pax9*$^{+/-}$ mutants compared to *Gbx2*$^{-/-}$ mutants (***$p < 0.0001$, Fisher's exact test). An abnormal thymus was always associated with an arch artery defect.

Table S5. Antibodies and probes used for immunostaining and in situ hybridisation.

Target	Catalogue Number	Species and Type	Supplier	Dilution
Primary antibody				
ERG1	ab92513	Rabbit monoclonal	Abcam	1:1000
Secondary antibody				
Donkey anti-rabbit IgG Alexa Fluor 594	A-21207	-	Thermo Fisher Scientific	1:200
Nuclear stain				
DAPI	H-1200	-	Vector Laboratories	-
RNAscope probes				
Pax9	454321-C2	Mouse	Advanced Cell Diagnostics	1:50
Tbx1	481911			Direct
Gbx2	314358			1:50
